# Impacts of Precipitation Variability on Carbon Flux Dynamics of Global Semi‐Arid Savannas

**DOI:** 10.1111/gcb.70954

**Published:** 2026-06-16

**Authors:** Laura Nadolski, Marieke Wesselkamp, Tarek El‐Madany, Markus Lange, Jacob Nelson, Arnaud Carrara, Aleksander Wieckowski, Anke Hildebrandt, Markus Reichstein, Sung‐Ching Lee

**Affiliations:** ^1^ Biogeochemical Integration Max‐Planck Institute for Biogeochemistry Jena Germany; ^2^ Faculty of Chemistry and Earth Science Friedrich‐Schiller University Jena Germany; ^3^ Fundacion Centro de Estudios Ambientales del Mediterráneo (CEAM) Valencia Spain; ^4^ Department of Physical Geography and Ecosystem Science Lund University Lund Sweden; ^5^ Department Computational Hydrosystems Helmholtz Centre for Environmental Research (UFZ) Leipzig Germany

**Keywords:** carbon cycle, drylands, ecohydrology, eddy covariance, land carbon sink, precipitation variability

## Abstract

With global warming, precipitation variability is increasing, affecting water availability and consequently ecosystem functioning and carbon uptake. Semi‐arid ecosystems are very sensitive to changes in water availability since both gross primary productivity (GPP) and ecosystem respiration (R_eco_) are strongly linked to it, contributing most to the interannual variability and trend of the global land carbon sink. However, their carbon‐dioxide (CO_2_) flux dynamics and specifically R_eco_ are not well understood and poorly represented in models. Here we compiled a long‐term global dataset of eight eddy‐covariance sites located in semi‐arid savannas to analyze the effects of precipitation variability on their net ecosystem exchange (NEE, with NEE = R_eco_—GPP). We assessed the effects of precipitation variability, represented by precipitation amount, frequency, intensity, and dry spell length, on both annual and seasonal CO_2_ fluxes. We used linear mixed effect models to identify relationships between the precipitation metrics, environmental factors, and CO_2_ fluxes. We further employed structural equation models to identify direct and indirect effects of precipitation variability on ecosystem CO_2_ fluxes in different phenological seasons. The results indicate that precipitation amount and intensity mostly lead to increasing GPP and R_eco_ on the annual scale, with different net effects on NEE depending on the sites. On the seasonal scale, both precipitation frequency and intensity explained more NEE variations than precipitation amount only, while the contribution of dry spell length was negligible. The three precipitation metrics together lead to highest model improvement. Across seasons, soil water content was the main mediator of precipitation impacts on CO_2_ fluxes, with air temperatures co‐dominating in the drydown season. Overall, this study shows that precipitation intensity, frequency, and their seasonal timing are crucial for explaining ecosystem CO_2_ flux dynamics of semi‐arid savannas. This can help improve predictions of the effects of increasing precipitation variability on the global carbon cycle.

## Graphical abstract

Semi‐arid ecosystems contribute most to the variability of the land carbon sink. They are water‐limited and therefore increased precipitation variability due to climate change has strong effects on their carbon cycle. We investigate the effects of precipitation amount, frequency, intensity and dry spell length on carbon fluxes of global semi‐arid savannas. We found different direct and indirect effects of these precipitation metrics across four distinct phenological seasons and could confirm that taking only precipitation amount into account is not sufficient for understanding seasonal carbon fluxes. Including precipitation amount, frequency and intensity helped explain most variance in seasonal carbon fluxes.

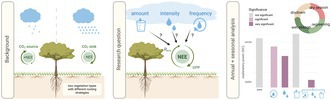

## 1 Introduction

Drylands cover around 40% of the global land surface, support about 60% of the global food production and encompass the largest fraction of grazing land on the globe (Maestre et al. 2022; Wang, Wang, et al. 2022; Wang and Collins 2024). In these water‐limited systems, precipitation strongly constrains vegetation functioning and productivity (Bernardino et al. 2025; Kannenberg et al. 2024; Wang and Collins 2024). This constraint is particularly consequential in semi‐arid regions, where small shifts in water supply drive large changes in carbon exchange, thereby making semi‐arid ecosystems the most important contributor to the interannual variability of the land carbon sink (Sitch et al. 2024). Depending on water availability and meteorological conditions, semi‐arid ecosystems can act as a carbon source or carbon sink, since both photosynthetic activity and respiration are limited by moisture (Del Grosso et al. 2018; Haverd et al. 2017; Piao et al. 2020). The global warming of the atmosphere intensifies hydrological processes and leads to an increasing variability in precipitation, further influencing this interannual variability (Douville et al. 2023; Feldman, Konings, et al. 2024; Pendergrass et al. 2017; Thornton et al. 2014; Zhang et al. 2021). Understanding the effects of increased precipitation variability on the carbon dynamics in semi‐arid ecosystems is therefore imperative to better understand the global land carbon sink.

With increasing precipitation variability, we expect less frequent but more intense precipitation events with longer dry spells in between (Feldman, Feng, et al. 2024; Giorgi et al. 2019; Knapp et al. 2017; Pendergrass et al. 2017). However, there is still a lack of knowledge on the underlying hydrological and physiological mechanisms of plant responses to these changing precipitation patterns (Feldman, Feng, et al. 2024). As a result, terrestrial biosphere and earth system models still fail to capture the effects on the global carbon cycle (Dai et al. 2024, 2025; Zhang, Wang, Zohner, et al. 2025), and especially respiration pulses are still widely underestimated (Metz et al. 2023; Nguyen et al. 2025).

Despite the same annual precipitation amount, the distribution of water among evapotranspiration, interception, runoff, and infiltration varies with changes in seasonal precipitation variability (Peng et al. 2013). High intensity increases infiltration due to both higher precipitation volumes and reduced interception (Lauenroth and Bradford 2012; Lian et al. 2022), leading to an increased rootzone moisture in deeper depths (Holdrege et al. 2023; Thomey et al. 2011). Contrastingly, increased rain intensity can also result in more runoff, when the soil is saturated or infiltration rates are exceeded (Short Gianotti et al. 2020). Longer dry spells in between precipitation events can cause extended periods of low soil moisture, high atmospheric aridity, and high solar irradiance due to less cloud coverage (Feldman et al. 2019; Grossiord et al. 2020). This leads to a drying in the plant–soil‐atmosphere continuum (Feldman et al. 2020; Feldman, Feng, et al. 2024), as well as decreased stomatal conductance, reduced photosynthesis, and slowed plant growth (Berdugo et al. 2020; Feldman et al. 2020). Therefore, changes in precipitation variability affect temporal distributions of root‐zone water storage changes (Feng et al. 2012; Peng et al. 2013) and consequently how soil microbes and different plant functional types with different rooting depths can access and use water. For example, grass species are very responsive to large moisture pulses, with response durations from several days to weeks (Guo et al. 2016; Huxman, Snyder, et al. 2004; Potts et al. 2019). This behavior is known as the pulse‐reserve paradigm (Noy‐Meir 1973), in which strong precipitation events cause dryland species to grow, store carbohydrates into reserves, and downregulate photosynthetic activity until the next precipitation event (Reynolds et al. 2004). Tree species can also temporally benefit from intense precipitation events, when water is pushed below the main grass rooting zone, favoring deeper‐rooted woody plants. However, on longer time scales dryland trees tend to reduce their growth with fewer, more intense precipitation events, since longer dry periods in between can lead to higher drought stress (Dannenberg et al. 2019; Feldman, Feng, et al. 2024; Wise and Dannenberg 2022). Therefore, the response of semi‐arid savannas (i.e., tree‐grass ecosystems) to changing precipitation patterns is particularly complex, since their two‐layered structure contains contrasting plant functional types with shallow versus deep roots and distinct water use strategies, competing for limited water resources (Whitley et al. 2017).

Furthermore, the timing of precipitation strongly modulates the carbon dioxide (CO_2_) flux responses, depending on the phenological status of the ecosystem. In the dry season, prolonged rain‐free intervals allow labile carbon and CO_2_ to accumulate. Subsequent rainfall produces large CO_2_ pulses via rapid microbial mineralization and physical displacement of CO_2_ by infiltrating water, leading to an increase in R_eco_. This is known as the Birch effect (Birch 1964; Borken and Matzner 2009; Nguyen et al. 2025; Roby et al. 2022; Unger et al. 2010; Xu et al. 2004). The CO_2_ pulse magnitude depends on event size and inter‐event spacing, antecedent soil moisture, temperature, solar radiation, humidity, and litter availability (Huxman, Snyder, et al. 2004; Manzoni et al. 2012; Moyano et al. 2013; Parton et al. 2012; Roby et al. 2020; Unger et al. 2012; Xu et al. 2004). After long droughts, pulses are larger and longer, whereas repeated rewetting depletes labile carbon and diminishes mineralization rates (Jarvis et al. 2007; Reichmann et al. 2013). Consequently, rain‐pulse responses often peak near the end of the dry season, when accumulated litter provides ample substrate (Hao et al. 2020). Precipitation variability modulates these dynamics by shaping drying–wetting cycles: low frequency prolongs dry intervals and tends to amplify rewetting respiration, whereas during the growing season with an active grass layer, repackaging rainfall into fewer, larger events can reduce soil CO_2_ efflux and lower cumulative respiration (Huxman, Snyder, et al. 2004; Roby et al. 2022).

Recent ecological studies ranging from single site to global remote sensing studies highlight that precipitation variability impacts vegetation functioning and ecosystem productivity (Feldman, Konings, et al. 2024; Liu et al. 2020; Wang, Liu, et al. 2022; Wang and Collins 2024; Zhang et al. 2022; Zhang, Wang, Zohner, et al. 2025). Available studies covering multiple sites using satellite data, mostly focus on the effects of changing interannual precipitation variability on productivity (Gherardi and Sala 2019; Hou et al. 2021; Hsu et al. 2012; Sasaki et al. 2023). Mostly, a positive effect of enhanced interannual precipitation variability on productivity is expected in dry ecosystems (Feldman, Feng, et al. 2024; Liu et al. 2020; Sasaki et al. 2023; Thomey et al. 2011; Zhang et al. 2022). However, these studies mostly neglect the effects on respiration even though they play a crucial role for the ecosystem CO_2_ balance (Nguyen et al. 2025). Dryland studies that take into account higher temporal resolution and also respiration processes, mostly focus on single sites, assessing the effects of changing seasonal precipitation variability on ecosystem CO_2_ fluxes (Arca et al. 2021; Delgado‐Balbuena et al. 2023; Guo et al. 2016; Hao et al. 2020; Liu et al. 2017; Peng et al. 2013; Post and Knapp 2020; Zhang et al. 2022).

Eddy‐covariance (EC) flux measurements provide insights into both ecosystem productivity and respiration (Baldocchi 2014, 2008), allowing for comprehensive and temporally highly resolved analyses of CO_2_ flux dynamics (Baldocchi 2020). However, flux measurements in semi‐arid regions are underrepresented, specifically long‐term in situ measurements remain very sparse (Jung et al. 2020). Savanna ecosystems, in particular, are an understudied dryland ecosystem type (Whitley et al. 2017). Most dryland studies which focus on the effects of changing precipitation patterns on the ecosystem carbon balance consider grasslands only (Arca et al. 2021; Chou et al. 2008; Collins et al. 2025; Legesse et al. 2021; Post and Knapp 2020; Ru et al. 2022; Sasaki et al. 2023; Zhang et al. 2022) or grasslands and shrublands (Biederman et al. 2018; Gherardi and Sala 2015, 2019). A study combining the data of multiple semi‐arid savanna sites in order to investigate effects of changing seasonal precipitation variability on the carbon fluxes of this type of ecosystem is still missing.

Here, we compile the first long‐term (10 years or more) in situ dataset from eight globally distributed EC sites in semi‐arid savanna ecosystems. We look into the net ecosystem exchange (NEE), describing the balance between CO_2_ uptake through photosynthesis (gross primary productivity, GPP) and release through ecosystem respiration (R_eco_, with NEE = R_eco_—GPP) (Baldocchi 2008). In this paper, we use the term “CO_2_ fluxes” to refer collectively to NEE and its component fluxes GPP and R_eco_.

Previous multi‐site studies represented precipitation variability using the inter‐annual variance of precipitation (Gherardi and Sala 2019; Hou et al. 2021; Sasaki et al. 2023) or wet day frequency alone (Feldman, Konings, et al. 2024). Here, we distinguish precipitation intensity, precipitation frequency and dry spell length additionally to precipitation amount to account for precipitation variability. We investigate the effects of these metrics on the ecosystem CO_2_ fluxes in different seasons to take into account the timing of precipitation and the phenological status of the ecosystem.

We aim to answer the following questions: (i) How do R_eco_ and GPP in semi‐arid savanna ecosystems respond to precipitation variability on the annual scale and consequently, how do fewer but more intense events affect their annual carbon balance (i.e., NEE)? (ii) Are precipitation frequency, precipitation intensity, and maximum dry spell length adequate metrics of precipitation variability to explain more variation in seasonal CO_2_ fluxes? Does the seasonal timing of precipitation matter? (iii) What are the direct and indirect effects of precipitation variability on the CO_2_ fluxes in different phenological seasons?

We hypothesize that (i) higher values of precipitation metrics can generally lead to increases in productivity, but also respiration of semi‐arid savannas, highlighting that timing of precipitation is crucial for determining which one overweighs. Secondly, we hypothesize that (ii) taking into account precipitation intensity, frequency, and dry spell length on top of precipitation amount, as well as seasonal timing of precipitation can help to better explain CO_2_ flux dynamics in semi‐arid savannas, since the temporal distribution of precipitation alters the root‐zone soil moisture distribution and therefore the accessibility of water by different plants. Lastly, we hypothesize that (iii) most effects of precipitation on CO_2_ fluxes are indirectly mediated by soil water content, with additional indirect effects through air temperature, radiation, and vapor pressure deficit.

By addressing these research questions, we aim to understand ecosystem processes in depth on the one hand, and draw general conclusions about the effects of changing precipitation patterns on the CO_2_ fluxes of global semi‐arid savannas on the other hand. Both will help to gain more insights into how global carbon dynamics might evolve under increasing precipitation variability in the future and tackle an important shortcoming in current terrestrial biosphere models.

## 2 Data

### 2.1 Site Selection

**FIGURE 1:**
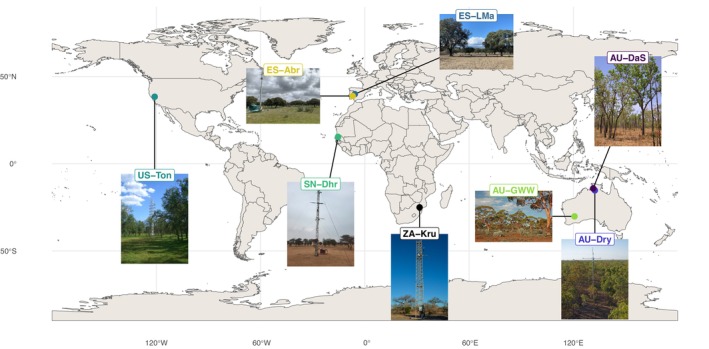
World map showing location and images of the eight savanna sites in our dataset: Majadas de Tiétar (ES‐LMa), Albuera (ES‐Abr), Tonzi Ranch (US‐Ton), Dahra (SN‐Dhr), Skukuza (Za‐Kru), Daly River Savanna (AU‐DaS), Dry River (AU‐Dry), and Great Western Woodlands (AU‐GWW). Photo sources: Australian sites (https://www.ozflux.org.au/monitoringsites/), ES‐LMa (Laura Nadolski), ES‐Abr (Laura Nadolski), US‐Ton (Dennis Baldocchi), and SN‐Dhr (Wieckowski et al. 2024). Map lines delineate study areas and do not necessarily depict accepted national boundaries.

To test the hypotheses and investigate the effects of precipitation dynamics on ecosystem‐atmosphere interactions, we compiled flux and environmental data from EC measurement sites located in savanna ecosystems across the globe (Figure 1). We chose sites that have a distinct wet and dry season and are not intensely affected by human activities. Additionally, they all have comparable vegetation structures and phenological cycles, facilitating the formulation of generalized assumptions and the definition of similar seasons across ecosystems. The sites feature a two‐layered vegetation composition, with a tree canopy cover < 40%, a negligible shrub layer (< 10%), and a predominantly annual grass layer. Additionally, we included only sites with a minimum of 10 years of data to enable reliable trend assessment.

The EC sites that comply with these criteria are: Majadas de Tiétar (ES‐LMa) and Albuera (ES‐Alb) in Spain, Tonzi Ranch (US‐Ton) in the United States, Dry River (AU‐Dry), Daly River savanna (AU‐DaS) and Great Western Woodlands (AU‐GWW) in Australia, Dahra (SN‐Dhr) in Senegal, and Skukuza (ZA‐Kru) in South Africa. The sites vary in mean annual precipitation (MAP), spanning from the relatively arid SN‐Dhr (MAP ~300 mm/year) to the more humid AU‐DaS (MAP ~1200 mm/year).

An overview of the sites, their metrics and basic vegetation characteristics is shown in Table 1.

**TABLE 1:** Overview of site characteristics.

FLUXNET ID	Country	Tree layer	Average tree height	Tree canopy cover (%)	MAP (mm)	MAT (°C)	Rain gauge type	Tip (mm)	Accuracy	Observation period	Main reference
AU‐DaS	Australia	Evergreen	16.4 m	10–30	1235	26.9	Tipping bucket rain gauge (HS Hyquest Solutions Pty. Ltd., CS700, Australia)	0.254	2.0% at 250 mm/h	2008–2022	Beringer et al. (2011)
AU‐Dry	Australia	Evergreen	12.3 m	10–30	844	27.0	Tipping bucket rain gauge (HS Hyquest Solutions Pty. Ltd., CS700, Australia)	0.254	2.0% at 250 mm/h	2010–2022	Beringer et al. (2011)
AU‐GWW	Australia	Evergreen	(up to over 20 m)	10–30	308	19.6	Tipping bucket rain gauge (Observator Instruments, RIM8000, Australia)	0.2	3.0% at 380 mm/h	2013–2022	Prober et al. (2023)
ES‐Abr	Spain	Evergreen	6.6 m	24	303	17.8	Tipping bucket rain gauge (Thies CLIMA precipitation transmitter, Germany)	0.1	3.0%	2016–2024	El‐Madany et al. (2020)
ES‐LMa	Spain	Evergreen	8.7 m	20	676	17.4	Weighing rain gauge (TRwS 514 precipitation sensor, MPS systém Ltd., Slovakia)	0.1	0.1% at 120 mm/min	2014–2024	El‐Madany et al. (2018)
SN‐Dhr	Senegal	Deciduous	5.2 m	3	321	28.6	Tipping bucket rain gauge (ARG100, EML, United Kingdom)	0.2	4.0% at 22 mm/h	2010–2022	Wieckowski et al. (2024)
US‐Ton	USA	Deciduous	14 m	40	599	16.6	Tipping bucket rain gauge (TE525, Texas Electronics, USA)	0.1	1.0% at 50 mm/h	2001–2023	Ma et al. (2016)
ZA‐Kru	South Africa	Deciduous	5–8 m	10–30	547	21.9	Tipping bucket rain gauge (TE525, Texas Electronics, USA)	0.1	1.0% at 50 mm/h	2001–2024	Kirton et al. (2009)

*Note:* MAP and MAT were calculated based on hydrological years included in the study, except for ZA‐Kru, where the time series had many gaps, here we took the values from the literature. Tip and accuracy refer to the rain gauge type. Data availability shows the years used for this study.

Abbreviations: MAP, mean annual precipitation; MAT, mean annual temperature.

### 2.2 Seasons

The selected savannas are characterized by a distinct dry and wet season. In between, there are two transitional seasons reflecting the phenological status of the grass layer: the drydown period of the grass layer and the regreening period that starts with the onset of rains before the main wet season (Hingerl et al. 2025; Nadolski et al. 2025). In summary, there are four seasons including wet season, drydown, dry season, and regreening.

The timing of these seasons varies from site to site according to regional synoptic systems, such as the West African monsoon (Hingerl et al. 2025), and also slightly from year to year due to the high interannual precipitation variability (Haverd et al. 2017). However, here we fixed the months in which the seasons typically occur across years to be able to identify the effect of changing precipitation patterns on the ecosystem carbon fluxes. To do this, we follow information according to literature and communication with site principal investigators. The detailed definitions of each season at each site can be found in the Table S1. Since the seasonality of precipitation is substantially changing during the recorded time period at AU‐GWW (communication with site teams), the season classification as done for the other sites was not possible here. Furthermore, we would like to point out that even though AU‐GWW was classified as savanna in FLUXNET, after communication with experts from Ozflux, we found that due to a lack of continuous grass layer, the ecosystem should rather be classified as a woodland. Nevertheless, we decided to keep the site in the annual analysis to showcase the effect of a missing continuous grass layer on the ecosystem CO_2_ fluxes.

### 2.3 Measurements and Data Processing

All sites have similar instrumentation to measure high‐frequency CO_2_ fluxes, including a three‐dimensional sonic anemometer along with an infrared gas analyzer. For sites including US‐Ton (Ma et al. 2023), AU‐Dry (Beringer and Hutley 2016a), AU‐DaS (Beringer and Hutley 2016b), AU‐GWW (Macfarlane et al. 2016), and SN‐Dhr (Wieckowski 2024), the data was publicly available and processed following the FLUXNET format (Pastorello et al. 2020). The data from ES‐LMa and ES‐Abr were collected and processed similar to the FLUXNET standards as described in El‐Madany et al. (2018) and (2020). For partitioning NEE into GPP and R_eco_, the night‐time partitioning method (Reichstein et al. 2005) was used at all sites, except at SN‐Dhr where the daytime method was used (Lasslop et al. 2010; Wieckowski et al. 2024). The raw flux data from ZA‐Kru was processed as described in Supporting Information S2. The downloaded dataset from AU‐Dry (Beringer and Hutley 2016a) showed some artefacts, and therefore we did not use the CO_2_ flux data (NEE, GPP, and R_eco_) from the periods 2013‐06‐10 until 2013‐10‐10 and 2015‐12‐28 until 2016‐02‐22. GPP and R_eco_ showed artefacts in 2011, so we removed the data from this complete year. Hence, the hydrological years 2011–2014 and 2016 and the affected seasons were removed from the analysis. The partitioned fluxes in the downloaded dataset from AU‐DaS (Beringer and Hutley 2016b) showed gaps in 2009, 2011 and 2017. Therefore, the hydrological years 2009–2012, 2017 and 2018 were removed from the analysis, as well as the respective seasons within these years. This leaves both sites (AU‐Dry and AU‐DaS) with eight complete site (hydrological) years.

In addition to the half‐hourly flux data, the following environmental variables were measured at all the sites: precipitation (P, mm), air temperature (Ta, °C), soil water content (SWC, %), soil temperature (Tsoil, °C), vapor pressure deficit (VPD, hPa) and incoming shortwave radiation (SW_in_, Wm^−2^). At SN‐Dhr, only photosynthetically active radiation (PAR) was available. We calculated SW_in_ from it as follows: SW_in_ [W m^−2^] = 0.482 * PAR [μmol m^−2^ s^−1^], assuming that PAR is on average around 45% of shortwave radiation (Howell et al. 1983) and converting it from μmol m^−2^ s^−1^ to W m^−2^. For the sites with soil measurements from multiple profiles (i.e., ES‐Abr and ES‐LMa), we used SWC measurements from the open pasture (i.e., exposed to the sun and not shaded by trees' shadows), as it constitutes around 80% of the overall area. Half‐hourly flux data and meteorological data were collected and processed as described in the respective site literature references (Table 1).

## 3 Statistical Analyses

We aggregated the half‐hourly data to daily, seasonal, and annual values to study the precipitation‐CO_2_ relationships on different scales. We defined four different precipitation metrics, namely precipitation amount, frequency, intensity and maximum dry spell length. First, we qualitatively investigated how the four precipitation metrics affect the balance between GPP and R_eco_ on the scale of hydrological years. Then we used linear mixed effect models (LMMs) to detect the importance of the different precipitation metrics in explaining NEE in different seasons, while additionally accounting for potential covariates. In the final step we designed structural equation models (SEMs) for each season showing the direct and indirect effects of precipitation metrics on GPP and R_eco_ as well as their respective importance. These steps are described in detail in the following sections.

### 3.1 Data Aggregation and Precipitation Metrics

We first aggregated the half‐hourly environmental and flux data to daily means, and in case of P to daily sums (P_*d*_).

Based on daily values we defined four metrics. First, the overall precipitation:

- Precipitation amount:
(1) $$P_{\text{amount}}=\sum_{i=1}^{n}P_{d}$$
  as the sum of daily precipitation amount over a certain number of days *n* [mm]. Next, the precipitation can be disintegrated into the frequency of rain days and average rainfall intensity, yielding two more metrics as follows:
- Precipitation frequency:
(2) $$P_{\text{frequency}}=\frac{n_{p2\mathrm{mm}}}{n}$$
  where *n*_p2 mm_ is the number of days with 2 mm or more of precipitation, and n as the number of days. We chose *n*_p2 mm_ to approximate “effective” precipitation days, since in dry ecosystems the water input from small precipitation events can sometimes evaporate really quickly and therefore will not be available to plants or microbes (McKellar and Crimmins 2025). For finding a threshold from which P is ecologically relevant, we tested for which thresholds P_frequency_ has a significant effect on NEE with a LMM (c.f. section 3.3 and details in S3).
- Precipitation intensity:
(3) $$P_{\text{intensity}}=\frac{P_{\text{amount}}}{n_{P}}$$
  With *n*_P_ being the number of days with precipitation (*p* > 0 mm). P_intensity_ describes the average daily precipitation depth over all days with precipitation [mm/d]. We chose *p* > 0 mm as a threshold here since we want to account for daily intensities of P. If there is no precipitation and accordingly no days with rain, we set P_intensity_ to “0”. Lastly, we defined a metric accounting for the length of dry spells in between precipitation events:
- Maximum dry spell length:
  Dryspell_max_ is the maximum number of days without precipitation in between days with effective precipitation (*n*_p2 mm_) within a certain time period [d]. If there is no precipitation event within a respective period (*n*_p2 mm_ = 0), then the number of days in this period is used for the dryspell_max_. If a dry spell crosses periods, it is attributed to the period at the end of the dry spell, to capture its ecological relevance.

All four metrics are hereafter referred to under the umbrella term of P metrics.

In the next step we computed annual values for hydrological years. We defined hydrological years starting in the month when the regreening season starts at the respective site, and then including the following wet season, drydown and dry season. In this way, the hydrological year differs in timing between locations, but includes the complete vegetative cycle of the annual grass layer (Luo et al. 2020; Nair et al. 2024). We calculated sums for the fluxes (NEE, GPP, R_eco_), P metrics, and means of the environmental variables (Ta, VPD, Tsoil, SWC, SW_in_). Hereafter we refer to those as annual values. Finally, we calculated aggregated values for each season at each site, in a similar manner as the annual values: P metrics as described, sums of the CO_2_ fluxes and means of the environmental variables.

Unfortunately, the data of ZA‐Kru presented too many gaps (Figure S2) to obtain robust seasonal or annual estimates and we had to discard it from further analysis. Since in AU‐GWW we could not define the phenological seasons as for the other sites, we only included this site in the analysis at the annual scale, with the hydrological year starting in March.

### 3.2 Linear Relationships

To understand the relationships between the precipitation metrics and annual NEE budgets, we used bivariate linear regressions and tested for significant relationships, with a significance level of *p* < 0.05. The analysis on the annual scale provides an overview of the relationships between different P metrics and CO_2_ fluxes across globally distributed savanna ecosystems and evaluates how annual precipitation patterns affect the ecosystems' carbon budget. To get deeper insights into the seasonal dynamics and also take into account other possible influencing factors of the CO_2_ flux dynamics such as air temperature and VPD, we used LMMs on the seasonal scale.

### 3.3 Linear Mixed Effect Models

We employed LMMs using the “lme4” package in R (Bates et al. 2015) to assess the impact of the P metrics on NEE across seasons. First, all predictor variables were standardized using *z*‐scores to ensure comparability of effect sizes and enhance numeric stability.

We then conducted a targeted nested model comparison fit by maximum likelihood to assess the relative explanatory value of the different P metrics for NEE. Site and year were included as nested random intercepts, since there are four seasons per hydrological year per site (i.e., multiple occurrences of the same year at the same site), but differing amounts of years available at each site. SWC modulates the relationship between NEE and the environmental variables in water‐limited ecosystems (Jia et al. 2020; Kannenberg et al. 2024; Xu et al. 2004) with a varying effect of SWC on NEE per site, considering the sites are characterized by diverse plant species with individual water use strategies and soil characteristics (Morgan et al. 2025). Therefore, we allowed random slopes for SWC in the models. We fit a baseline model without fixed effects (m0), then added precipitation metrics as fixed effects stepwise: one metric (m1), two metrics (m2), three metrics (m3), and four metrics (m4). To verify that inferences were not dependent on entry order, we evaluated all 24 possible entry orders (permutations) of the four precipitation metrics. Incremental contributions of added metrics were evaluated using likelihood‐ratio tests (ANOVA) and changes in Akaike information criterion (AIC). Model diagnostics, including checks for singular fits, were monitored. Across all 24 permutations, dryspell_max_ provided negligible unique explanatory power; therefore, it was not used for the remaining analyses.

Secondly, we built LMMs for investigating relationships between P metrics, environmental variables and GPP and R_eco_, to gain more insights into ecosystem processes and to find out if they differ across seasons. Since the P metrics are collinear by definition, we conducted the analysis by fitting two separate models to the seasonal data aggregates for each P metric, with the remaining model structure and predictors being equal across P models.

While our main approach to model selection is founded on mechanistic theory, our goal was to estimate effect sizes for P metrics. To mitigate collinearity, we first used Pearson correlation coefficient to identify redundant predictors (correlation coefficient > 0.7) (Figure S4) (Dormann et al. 2013). We identified a strong positive correlation between Ta and Tsoil. Based on their similarity we decided to keep only one of them per model. Since Tsoil is an important predictor of R_eco_ (Conant et al. 2000; Richardson et al. 2012), we kept Tsoil for the R_eco_ model. For the GPP model we excluded Tsoil, and retained Ta as the representative variable. While VPD strongly affects photosynthesis and surface conductance in semi‐arid ecosystems, R_eco_ is less affected (Roby et al. 2020), and we therefore discarded VPD from the R_eco_ model. The remaining fixed effects were the same across models: The effect of Tsoil on R_eco_ depends on water availability (SWC) (Xu et al. 2004) and is hence introduced as interaction term (SWC*Tsoil) in the LMMs. SW_in_ has a distinct effect on the ecosystem CO_2_ fluxes as it is a driver of photosynthesis and enhances photochemical degradation enhancing availability of labile carbon that can be respirated (Gliksman et al. 2017), and is therefore kept in the models despite its moderately strong correlation with Ta and SWC (Figure S4).

To evaluate the effects of the P metrics on NEE, we constructed four parallel LMMs, each incorporating one of the P metrics (Equations 4 and 5). We expected P metrics to vary with season and introduced this as an interaction term (season*P). The error structure remained the same as described for the targeted nested model comparison. However, for the R_eco_ models we found the variation of SWC over site/year to be marginal or zero, meaning that the slope of the SWC‐R_eco_ relationship does not vary significantly across sites/years. We therefore did not allow random slopes for SWC in the R_eco_ models. To ensure model convergence while maintaining the maximum random effects structure, we selected our final models as follows:

(4) $$\mathrm{GPP}\sim \mathrm{Ta}+\mathrm{SWC}+{\mathrm{SW}}_{\text{in}}+\mathrm{VPD}+\text{season}*P+\left(\mathrm{SWC}\;|\text{Site}/\text{year}\right)$$

(5) $$R_{\mathrm{eco}}\sim \text{Tsoil}*\mathrm{SWC}+{\mathrm{SW}}_{\text{in}}+\text{season}*P+\left(1\;|\text{Site}/\text{year}\right)$$

where P ∈{P_amount_, P_frequency_, P_intensity_}. Therefore, in total there are 2 × 3 = 6 LMMs. The LMMs were fitted with maximum likelihood to ensure unbiased fixed effects. For significance testing, we used the default Satterthwaite's test type III from the “lmerTest” package in R (Kuznetsova et al. 2017). In a post hoc analysis with the “emmeans” package (Lenth and Piaskowski 2026), we calculated marginal slopes and respective standard errors of P metrics and inferred their significance for all LMMs.

We examined the random slopes and intercepts visually for adherence to a normal distribution to diagnose the models. Additionally, we employed Shapiro–Wilk tests to evaluate the normality of the residuals and used visualization to assess if the remaining linear model assumptions (i.e., homogeneity and linearity of residuals) were met.

### 3.4 Structural Equation Models

SEMs were employed to reveal whether the observed NEE responses are attributable to coordinated changes in GPP, R_eco_, or compensatory shifts between them. Here we built SEMs which treat GPP and R_eco_ as endogenous variables and relate them to the same set of environmental drivers considered in the LMMs, enabling partitioning of direct and indirect effects on each component and clearer attribution of mechanisms. We designed SEMs using the R package “piecewiseSEM” (Lefcheck 2016) to perform a confirmatory path analysis to test causal relationships between precipitation metrics, environmental variables and CO_2_ fluxes in different seasons.

SEMs address complex multivariate relationships among a set of interconnected variables. They are probabilistic models bringing together various predictor and response variables in one causal network (Lefcheck 2016). One variable can serve as a response in one path, and as a predictor in a different path. Therefore, SEMs are able to test and quantify indirect effects and cascading effects that would not be recognized by a single model (Grace 2006) and are a useful tool to understand ecological processes (Hu et al. 2024; Huang et al. 2024; Lange et al. 2015, 2021).

Confirmatory path analysis, a specific type of SEM, allows for the fitting of a broad range of distributions and sampling designs. It further is able to fit smaller datasets than the traditional maximum‐likelihood SEM, since it estimates equations separately instead of globally (Lefcheck 2016).

We built one SEM for each season, since the LMMs showed that importance of precipitation metrics differs across seasons and we also expected different CO_2_ flux driver importance in different seasons (Ma et al. 2016; Nadolski et al. 2025). We started for each season from the same baseline model (Figure S6) based on physical principles and pre‐existing knowledge about relationships between precipitation, VPD, Ta, SW_in_ and SWC and the CO_2_ flux in semi‐arid savannas. As response variables we used GPP and R_eco_ in order to gain in‐depth understanding of ecosystem processes. In general, we kept the error structure of the LMM's, that is, site was fitted as a random factor to account for the spatial dependence of the measurements. However, we do not include year in the error structure, since we applied separate SEMs for each season. Furthermore, we allowed random slopes for SWC in the paths that explain GPP, since the soil water availability modulates the GPP—environmental variable relationships. Starting from the baseline model, we then used Shipley's test of directed separation to ensure that there are no missing relationships between unconnected variables in our basis set (Lefcheck 2016; Shipley 2000). We further used Fisher's C as a diagnostic of model performance and to test if the model adequately represents the data (Lefcheck 2016).

## 4 Results

### 4.1 Relationships Between Annual CO_2_ Budgets and Precipitation

**FIGURE 2:**
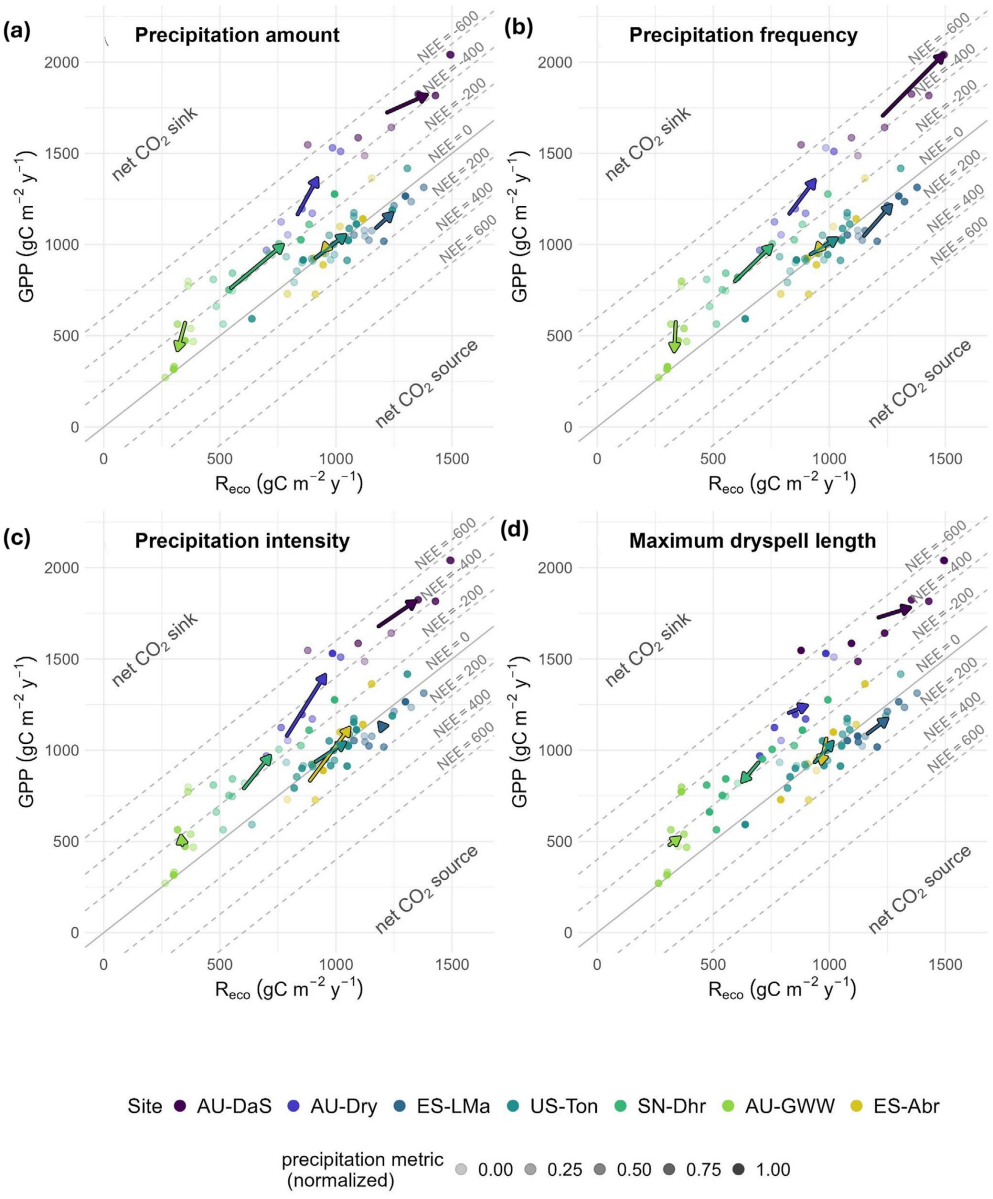
Balances between gross primary (GPP) and ecosystem respiration (R_eco_) on the scale of hydrological years across the sites (colors, from wettest to driest):Dahra (SN‐Dhr) Daly River Savanna (AU‐DaS), Dry River (AU‐Dry), Majadas de Tiétar (ES‐Lma), Tonzi Ranch (US‐Ton), Dahra (SN‐Dhr), Great Western Woodlands (AU‐GWW) and Albuera (ES‐Abr). The intensity of the color indicates the normalized precipitation metric per site for (a) precipitation amount, (b) precipitation frequency (rain days > 2 mm/number of days), (c) precipitation intensity (precipitation amount/number of rain days), and (d) maximum dry spell length. Arrows show from the average flux of the drier half of the years (split at the median value) to the average flux of the wetter half of the years to indicate the precipitation metric‐CO_2_ flux relationship. A positive net ecosystem exchange (NEE) indicates that the ecosystem acts as a carbon source, a positive NEE indicates a carbon sink.

Examining the relationships between annual CO_2_ fluxes and P metrics (Figure 2), the results show similar patterns across sites, with a few exceptions.

At many sites (ES‐LMa, SN‐Dhr, US‐Ton), both GPP and R_eco_ increase similarly with increasing P_amount_ (Figure 2a). At SN‐Dhr, the relationships of both GPP and R_eco_ are significantly positive (Figure S7a,b). Contrastingly, at AU‐DaS and ES‐Abr R_eco_ is enhanced more than GPP with increased P_amount_, shifting the ecosystem towards CO_2_ neutral or slight source at ES‐Abr, and towards a smaller CO_2_ sink at AU‐DaS. Nevertheless, AU‐DaS shows a significantly positive relationship between GPP and P_amount_ (Figure S7a), though outweighed by R_eco_. AU‐GWW shows a contrasting relationship to the other sites. With increasing P_amount_, both component fluxes decrease but GPP drops more rapidly than R_eco_.

With increasing P_frequency_, GPP increases more than R_eco_ at most sites (AU‐DaS, AU‐Dry, ES‐LMa, SN‐Dhr), turning the ecosystem into a stronger CO_2_ sink, or more towards CO_2_ neutral (ES‐LMa) (Figure 2b). At AU‐DaS this GPP‐P_frequency_ relationship is significantly positive (Figure S7c). At US‐Ton, even though the effect of P_frequency_ on GPP is significantly positive, the effects on GPP and R_eco_ are both positive and cancel out on the annual scale. At ES‐Abr, we observe a similar pattern to the relationship with P_amount_. R_eco_ increases slightly more with increasing P_frequency_, turning the ecosystem into CO_2_ neutral or a weak source. AU‐GWW again shows a contrasting relationship to the other sites: GPP decreases with increasing P_frequency_, while R_eco_ does not change much. The ecosystem accordingly behaves as a smaller CO_2_ sink.

The effect of P_intensity_ is stronger on GPP than on R_eco_ at SN‐Dhr, AU‐Dry and ES‐Abr, turning the ecosystems into stronger CO_2_ sinks with increasing P_intensity_ (Figure 2c). At SN‐Dhr, both the R_eco_‐P_intensity_ as well as GPP‐P_intensity_ relationships are significantly positive, where the GPP increase occurs to be stronger than that of R_eco_ (Figure S7e). At US‐Ton, both component flux‐P_intensity_ relationships are also significantly positive; however, R_eco_ increases slightly more (Figure S7e). This leads to a slight increase in NEE (i.e., weaker CO_2_ sink) with increasing P_intensity_ at US‐Ton (Figure 2c). Similarly, at ES‐LMa, R_eco_ decreases slightly more with P_intensity_ compared to GPP, leading to a more positive NEE. At AU‐DaS, both component fluxes increase to a similar degree with increasing P_intensity_. AU‐GWW is again the exception, with increasing GPP following higher P_intensity_ but R_eco_ not responding to it.

With reduced dryspell_max_ (i.e., smaller periods between precipitation events), GPP and R_eco_ increase similarly at AU‐GWW, ES‐LMa and US‐Ton, resulting in a stable NEE (Figure 2d). At AU‐Dry and AU‐DaS, R_eco_ increases more than GPP, leading to slightly weaker CO_2_ sinks. Contrastingly, at SN‐Dhr and ES‐Abr, GPP decreases more than R_eco_ with increasing dryspell_max_, leading to a stronger CO_2_ release and smaller CO_2_ sink.

### 4.2 Impact of Precipitation Variability and Environmental Variables on CO_2_ Fluxes Across Sites

**FIGURE 3:**
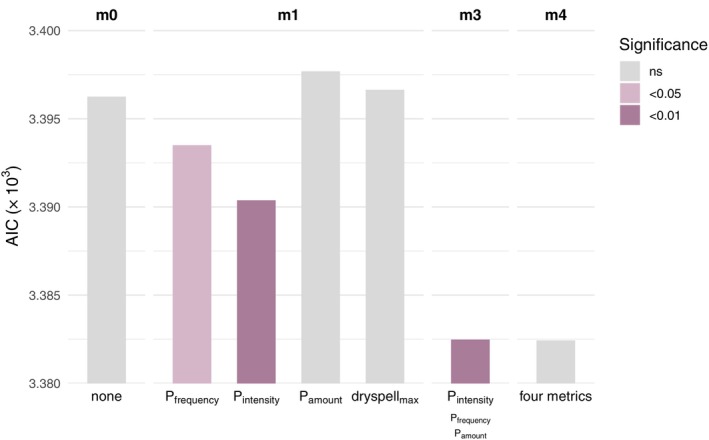
Akaike information criterion (AIC) of selected models from the targeted nested model comparisons. Colored bars show the significant improvement of the model compared to the antecedent model containing one precipitation metric less, tested with Analysis of Variance (ANOVA). P_frequency_ stands for precipitation frequency, P_intensity_ for precipitation intensity, P_amount_ for precipitation amount and dryspell_max_ for maximum dry spell length between precipitation events. Non‐significant improvements are marked with “ns” (grey). The model m0 contains no precipitation metric, m1 one precipitation metric, m2 two precipitation metrics, m3 three precipitation metrics and m4 all four.

The targeted nested model comparisons (maximum likelihood fits) show that P_intensity_ and P_frequency_ consistently improves model fit, whenever they were added (Figure 3 and Figure S5). In contrast, P_amount_ provides incremental improvement in about half of the additions, and dryspell_max_ is almost never informative (22/24 non‐significant) (Figure S5). When including P_intensity_ or P_frequency_ first, they improve model fit over the random‐effects–only baseline (*p* ≈ 0.005 and 0.029, respectively), whereas P_amount_ and dryspell_max_ do not (*p* ≈ 0.452 and 0.204, Figure S5). The model including all four predictors has the lowest AIC (3382.4 vs. 3396.3 for the null), supporting a contribution ranking of P_intensity_ ≈ P_frequency_>P_amount_ >> dryspell_max_. The 4‐metric model has the best AIC ≈ 3382.44, being only ~0.05 lower than the best 3‐metric model, which means an inconsequential difference, that does not significantly improve the model. As the incremental information from dryspell_max_ is marginal to non‐existent, we exclude dryspell_max_ from subsequent analyses. We would like to highlight that including P_frequency_, P_intensity_, and P_amount_ together significantly improves explaining variations in NEE (i.e., the best model performance of the ones including three metrics), whereas P_amount_ only proves to be not sufficient. Note that all models show singular random‐effects fits, so fixed‐effect inferences should be interpreted cautiously. Model diagnostics and *p*‐values of all models from the 24 sequences are provided in Figure S5.

The LMMs (Equations 4 and 5) give insights into the relationships between CO_2_ component fluxes and P metrics in different seasons, but also into the effects of other environmental covariates. The relationships between P metrics and CO_2_ component fluxes are season‐dependent.

**FIGURE 4:**
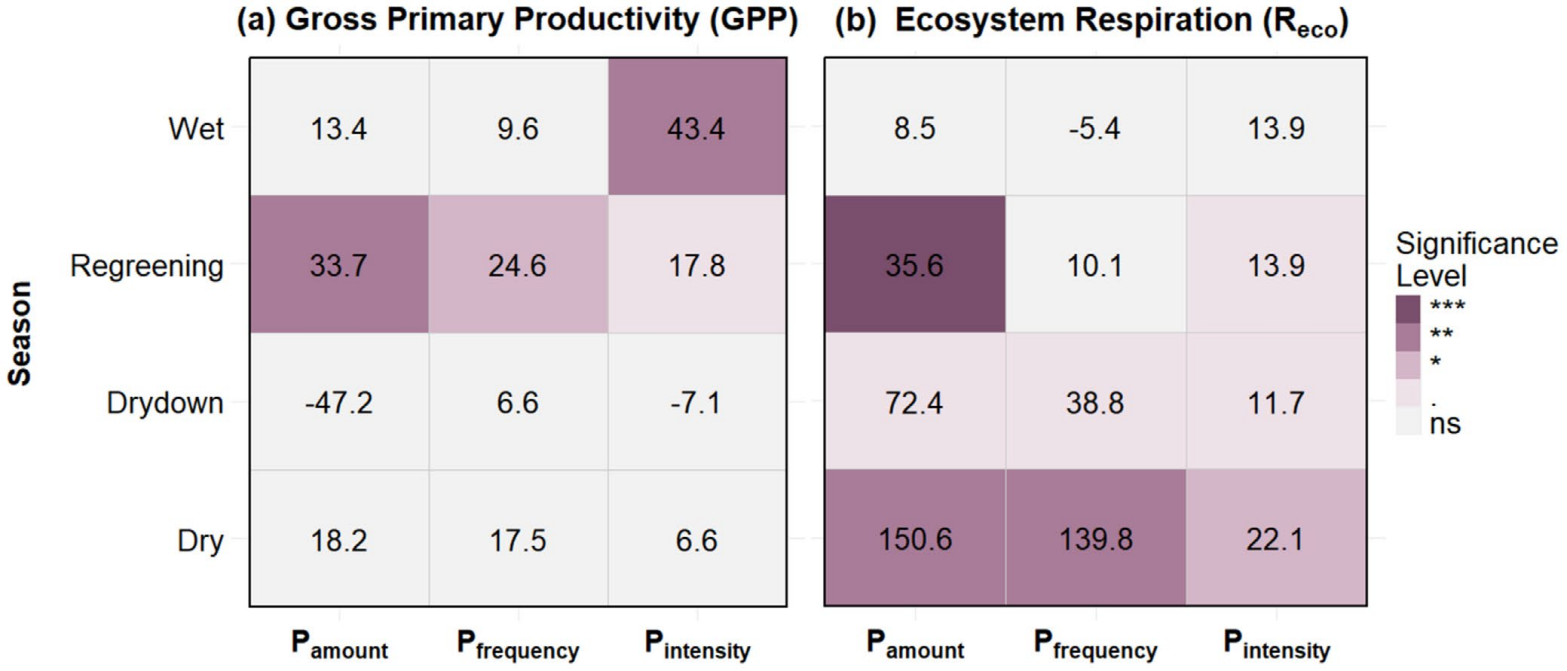
Effect sizes of precipitation amount (P_amount_), precipitation frequency (P_frequency_) and precipitation intensity (P_intensity_) on gross primary productivity (GPP) and ecosystem respiration (R_eco_) in defined phenological seasons (wet season, regreening, drydown and dry season). Individual effect sizes (simple slopes) were derived post hoc from linear mixed effect model interaction estimates. They are coloured by significance (*** stands for *p*‐values < 0.001, ** for *p*‐values < 0.01, * for *p*‐values < 0.05, . for *p*‐values < 0.1) which, beyond the magnitude of the estimated effects, indicate estimation certainty. These estimates show season‐dependent relationships between different metrics of precipitation variability and component fluxes (GPP and R_eco_).

The results of the GPP models show that the effects of the three P metrics on GPP are mainly significant in the regreening season and in the wet season, whereby P_amount_ shows the highest significance in the regreening season and P_intensity_ in the wet season (Figure 4, Table S9.1). In the regreening season, all P metrics are significant with the strongest effect of P_amount_ and the weakest of P_intensity_. In the drydown season the models show negative effects of P_amount_ and P_intenisty_ on GPP while positive in all other seasons. The negative effect of P_amount_ in the drydown season is large, but its standard error is too large to estimate it with certainty (Table S9.1).

The R_eco_ models indicate that all P metrics have positive effects across seasons, except P_frequency_ in the wet season. P_amount_ has a very strong significant effect on R_eco_ in the dry and regreening season, and weaker in the drydown (Figure 4, Table S9.2). P_frequency_ has most significance and the strongest effect size in the dry season. It is also significant in the drydown season but with a weaker and less significant effect. P_intensity_ has also the strongest and most significant effect in the dry season, and less strong effects in the drydown and regreening season. In the wet season, no P metric has a significant effect on R_eco_. The effects of P metrics on R_eco_ differ significantly between seasons (Table S8a–f, Figure S8).

In all three LMMs, GPP increases with SW_in_ and decreases with Ta (Table S8a,c,e). R_eco_ increases with SWC and SW_in_, but decreases with Tsoil in each model and Tsoil and SWC show a negative interaction. (Table S8b,d,f).

Model fit performances of the GPP and R_eco_ LMMs (Equations 4 and 5) are very similar across P metrics for GPP (AIC ≈ 3202.6 for P_amount_, 3209.3 for P_frequency_, and 3198.5 for P_intensity_), with P_intensity_ being marginally favored. For R_eco_, the model fit modestly favors P_amount_ (AIC ≈ 2938.6) over P_frequency_ (≈2951.2) and P_intensity_ (≈2952.9) (Tables S8a–f).

Random‐effects patterns are consistent across models. For GPP, between‐site variability in mean GPP is substantial as the intercept standard deviation (SD) is 76–86, with smaller variations among years within sites (SD ≈ 26–29). The SWC slope varies widely across sites (SD ≈ 62–79) and more modestly across years within sites (SD ≈ 18–20), indicating heterogeneous SWC–GPP relationships. For R_eco_, between‐site variability in mean R_eco_ is likewise substantial (Site intercept SD ≈ 50–56), with smaller variation among years within sites (SD ≈ 17–21) (Table S8a–f).

### 4.3 Direct and Indirect Effects of Precipitation on GPP and R_eco_ Across Seasons

**FIGURE 5:**
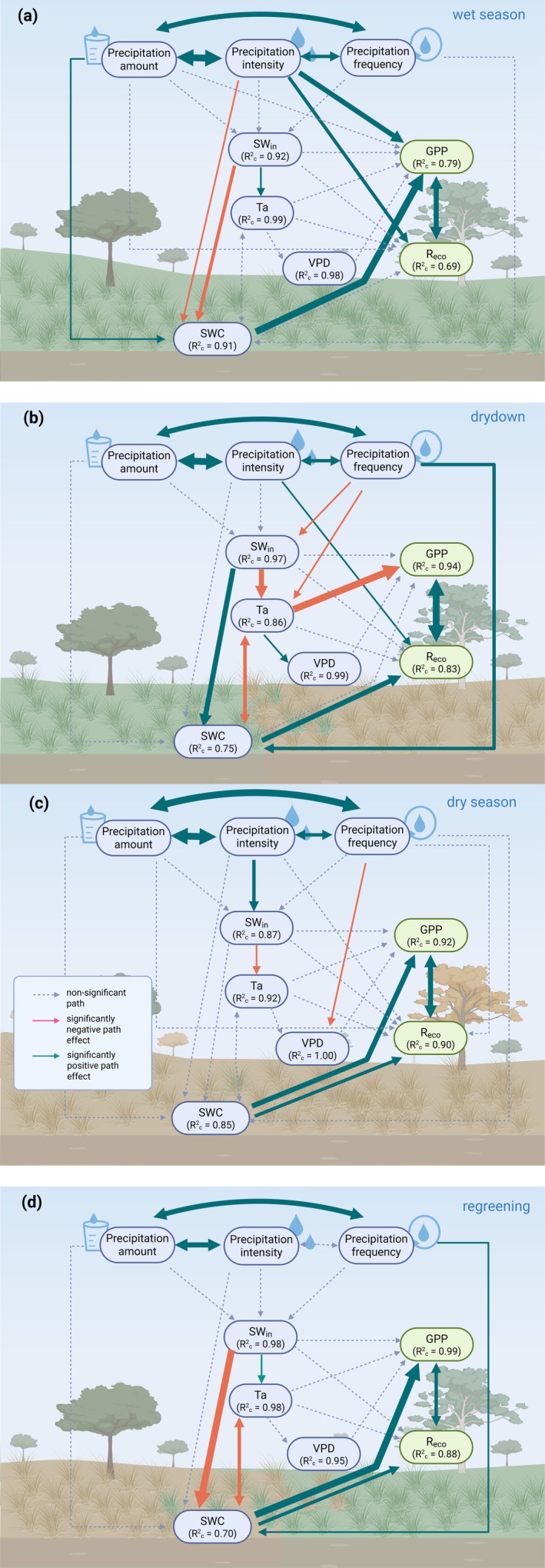
Structural equation models identifying direct and indirect effects of three precipitation metrics on gross primary productivity (GPP) and ecosystem respiration (R_eco_). Subplots show specific models for each season: (a) wet season, (b) drydown season, (c) dry season, and (d) regreening season. Green solid arrows represent significant positive path effects and pink solid arrows significant negative path effects (*p* < 0.05). Double arrows show intercorrelations and dashed arrows show non‐significant paths. *R* ^2^_*c*_ represents conditional coefficient of determination (*R* ^2^), which shows the proportion of variance explained by both fixed effects and random effects. The arrow widths represent the standardized path effects (four bins for arrow widths), with wider arrows showing stronger effects. Path coefficients and model diagnostics of all models (Fisher's *C*) can be found in Supporting Information S10. The plots were created with BioRender.

The SEMs indicate season‐specific direct and indirect path effects on GPP and R_eco_ with SWC as a dominant mediator in the regreening and wet seasons, and Ta as additional key mediator in the drydown season (Figure 5).

In the wet season (Figure 5a), P_intensity_ appears to be crucial for CO_2_ flux dynamics, with SWC as the central mediator. While P_amount_ increases SWC, with a standardized effect size (*β*std) of 0.32, SW_in_ (*β*std = −0.55) and P_intensity_ (−0.21) reduce SWC. In consequence, higher SWC increases GPP (1.00). SW_in_ increases Ta (0.19), but Ta, VPD and SW_in_ had no significant direct effects on the CO_2_ fluxes on the seasonal scale. P_intensity_ has a positive effect on both R_eco_ (0.50) and GPP (0.77), with a stronger effect on GPP. Residual GPP and R_eco_ are positively correlated, and the precipitation metrics were strongly intercorrelated (all *p* < 0.001). The wet season SEM showed good global fit (Fisher's C = 20.5, degrees of freedom (df) = 22, *p* = 0.6) and high explained overall variance in the response variables, when considering both random and fixed effects (conditional *R* ^2^_*c*_: GPP = 0.79, R_eco_ = 0.69, SWC = 0.91) (Figure 5a, Table S10.1).

During the drydown season (Figure 5b), the SEM identifies Ta and SWC as key mediators. The SEM indicates that P_frequency_ reduces SW_in_, with a standardized effect size of −0.13, while P_intensity_ (0.10) and P_amount_ (−0.11) had no significant effect on it. Both SW_in_ (0.49) and P_frequency_ (0.43) increase SWC. SW_in_ (−0.58) and P_frequency_ (−0.24) both decrease Ta, and Ta increases VPD (0.17). In turn, GPP is directly decreased by Ta (−0.79), while effects of VPD (−0.32), SW_in_ (0.36), and SWC (0.29) are not significant. R_eco_ increases with SWC (0.46) and P_intensity_ (0.18), with a marginal negative effect of Ta (−0.45, *p* = 0.07). Residual GPP and R_eco_ are positively correlated (*r* = 0.81), and Ta and SWC are negatively correlated (*r* = −0.32). Precipitation metrics are strongly intercorrelated (all *p* ≤ 0.001). The drydown SEM shows good global fit (Fisher's C = 22.79, df = 26, *p* = 0.65) and high explained variance in key responses (*R* ^2^_*c*_: GPP = 0.94, R_eco_ = 0.83, SWC = 0.75) (Figure 5b, Table S10.2).

In the dry season (Figure 5c), the SEM indicates low ecosystem activity with few significant paths. The SEM results suggest that higher P_intensity_ leads to an increase in SW_in_ (0.22) while P_amount_ tends to reduce it (−0.23, *p* = 0.07) and P_frequency_ shows no effect. No precipitation metric significantly affects SWC. Ta decreases with higher SW_in_ (−0.20). P_frequency_ reduces VPD (−0.02). GPP is directly increased by SWC (0.61), while effects of VPD (−0.30), SW_in_ (0.09), and Ta (−0.04) are not significant; R_eco_ increases with SWC (0.49), with no significant effects of Ta (−0.29), SW_in_ (−0.13), or any precipitation metric. Residual GPP and R_eco_ are positively correlated, and precipitation metrics were strongly intercorrelated (all *p* < 0.001). The dry‐season SEM shows adequate global fit (Fisher's C = 22.06, df = 22, *p* = 0.46) and high explained variance in the response variables (*R* ^2^_*c*_: GPP = 0.92, R_eco_ = 0.90, SWC = 0.85) (Figure 5c, Table S10.3).

During the regreening season (Figure 5d), the SEM identifies SWC as a central mediator, and the pathways resemble the wet season; however, without direct effects of precipitation on CO_2_ fluxes. The SEM indicates that SWC is jointly controlled by radiation and precipitation: SW_in_ strongly decreases SWC (−0.79), while P_frequency_ increases it (0.23). Consequently, GPP is directly increased by SWC (1.15), with no significant direct effects of VPD, SW_in_, or Ta. R_eco_ increases with SWC (0.44). Ta increases with SW_in_ (0.21). Residual GPP and R_eco_ are positively correlated, and Ta and SWC are negatively correlated. Precipitation metrics are generally intercorrelated (P_amount_–P_frequency_ and P_amount_–P_intensity_, both *p* < 0.001), whereas the P_intensity_–P_frequency_ correlation is not significant. The regreening SEM shows good global fit (Fisher's C = 42.82, df = 30, *p* = 0.06) and substantial explained variance in key response variables (*R* ^2^_*c*_: GPP = 0.99, R_eco_ = 0.88, SWC = 0.70) (Figure 5d, Table S10.4).

**FIGURE 6:**
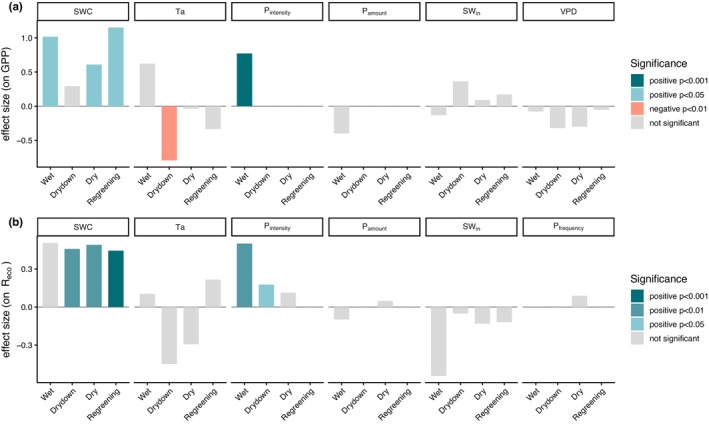
Standardized direct path effect sizes of soil water content (SWC), air temperature (Ta), precipitation intensity (P_intensity_), precipitation amount (P_amount_), precipitation frequency (P_frequency_), shortwave incoming radiation (SW_in_) and vapor pressure deficit (VPD) on (a) gross primary productivity (GPP) and (b) ecosystem respiration (R_eco_) from structural equation models across phenological seasons.

Across seasons, direct effects on GPP are dominated by SWC and Ta, with clear seasonal contrasts (Figure 6). SWC showed the strongest positive direct effects on GPP, peaking during regreening. The only direct effect of P metrics on GPP is found in the wet season for P_intensity_, indicating they act mainly indirectly via SWC. In contrast, Ta exerted a pronounced limiting effect on GPP in the drydown season, while direct effects of SW_in_ and VPD on GPP were generally small and inconsistent across seasons. For R_eco_, SWC consistently had positive direct effects (moderate to strong depending on season), whereas direct effects of Ta and SW_in_ were weak and seasonally variable. Only P_intensity_ showed direct positive effects in the wet season and drydown season.

## 5 Discussion

In this study we defined multiple precipitation metrics to approximate precipitation variability and studied their effects on CO_2_ fluxes on different time scales. We found that on the annual scale, their effects on the component fluxes are comparable in direction but partly differ in their effects on NEE among sites. However, our seasonal analysis suggests that independent of the site, there are different seasonal effects of precipitation variability on CO_2_ fluxes. P_frequency_, P_intensity_ and P_amount_ together provide the highest explanatory power for NEE. This indicates that deriving ecologically informed variables from precipitation records (i.e., feature engineering) can help to better represent the mechanisms by which water pulses regulate NEE. In the following sections, we discuss the ecosystem processes underlying our results.

### 5.1 Similar Effects of Precipitation Metrics on Annual CO_2_ Budgets Across Sites

Climate projections predict higher event intensity and fewer events with longer dry spells in between, especially in dryland areas (Feldman, Feng, et al. 2024; Giorgi et al. 2019; Pendergrass et al. 2017). Ecological models suggest that GPP and above‐ground biomass mostly increase under fewer, more intense precipitation events (Baudena et al. 2007; Guan et al. 2014; Zhang et al. 2016). Our results suggest that fewer, larger events can also reduce sink strength, but not uniformly; where they improve deep recharge accessible to vegetation, annual GPP can outpace R_eco_ and maintain or strengthen sinks. Where runoff or respiration dominates, sinks will weaken.

On the annual scale, we mostly identified positive effects of enhanced precipitation metrics (wetter conditions) on GPP and R_eco_ across semi‐arid savannas sites (Figure 2). At several sites (ES‐LMa, SN‐Dhr and US‐Ton), wetter years (higher P_amount_) increased both GPP and R_eco_ similarly, yielding limited net changes in NEE (Figure 2). At AU‐DaS and ES‐Abr, R_eco_ increased more than GPP as P_amount_ rose, weakening the sink or approaching CO_2_ neutrality. This likely reflects a greater stimulation of heterotrophic respiration when there are large water inputs into the system, increasing substrate diffusion and microbial activity (Biederman et al. 2018; Metz et al. 2023). At AU‐DaS, this is surprising since it is the wettest site, located in a more subtropical area. We suggest that here possible energy/radiation constraints caused by cloudier, cooler conditions in wet years cap GPP gains while R_eco_ continues to rise.

More frequent rainfall increased GPP more than R_eco_ at several sites (AU‐DaS, AU‐Dry, ES‐LMa, SN‐Dhr), strengthening the annual CO_2_ sink or shifting from source towards neutrality. Frequent, smaller events sustain shallow‐soil moisture, which aligns with the fine‐root distribution of grasses, prolonging active photosynthetic periods (Liu et al. 2017; Zhang, Biederman, Pierce, et al. 2025; Zhang et al. 2020). Reduced occurrence of long desiccation periods diminishes extreme moisture stress and canopy downregulation, supporting cumulative GPP (Zhang et al. 2020). R_eco_ gains are smaller where short wet‐ups create fewer large substrate flushes, dampening microbial respiration responses (Jarvis et al. 2007; Roby et al. 2022) compared to GPP.

With more intense precipitation events, stronger sinks arose at SN‐Dhr, AU‐Dry, ES‐Abr. More intense events likely increased infiltration depth and allowed moisture access for deeper roots and woody components, extending productive periods for both trees and grasses (Kulmatiski and Beard 2013; Post and Knapp 2020). At US‐Ton, ES‐LMa, and AU‐DaS, R_eco_ increased slightly more than GPP with more intense events, increasing NEE. Where R_eco_ rises similarly with or more than GPP, intense events may lead to stronger microbial respiration, or increase runoff that limits GPP gains.

Shorter dry spells had site‐dependent effects on CO_2_ fluxes. At AU‐GWW, ES‐LMa, and US‐Ton, they increased both GPP and R_eco_ by similar amounts, leaving NEE roughly unchanged. This is consistent with water‐limited systems where frequent small wettings sustain leaf water status and photosynthesis while also fueling microbial activity; rapid plant recovery (e.g., shallow‐rooted grasses/annuals) likely keeps pace with recurring respiration pulses, preventing a net loss of sink strength. In contrast, at AU‐Dry and AU‐DaS, R_eco_ increased more than GPP, weakening sinks. This pattern points to classic Birch‐type rewetting pulses and substrate mobilization that disproportionately boost heterotrophic respiration (Birch 1964; Huxman, Cable, et al. 2004; Moyano et al. 2013). At SN‐Dhr and ES‐Abr, shorter dry spells decreased both component fluxes, with a larger reduction in GPP, also weakening sinks. This could reflect frequent, small events that moisten only the surface, failing to recharge deeper layers needed for plant uptake while still sustaining some microbial turnover.

AU‐GWW, one of the driest sites, represents a boundary case in our dataset. Unlike the other sites, increases in P_amount_ and P_frequency_ are associated with lower GPP and show no strong relationship with R_eco_ (Figure S7). Consequently, NEE becomes lower with increasing precipitation, which is unusual for a strongly water‐limited system (MAP < 300 mm year^−1^). The site is located in the Great Western Woodlands in Southwestern Australia (Figure 1), where vegetation is dominated by open eucalypt woodland with occasional Acacia (*mulga*) patches. A continuous grass layer is largely absent, and extensive areas of bare red‐clay soil are present (Prober et al. 2023). Consequently, classic rain‐pulse effects on R_eco_ and pulse–reserve dynamics are likely muted relative to the other ecosystems, and CO_2_ flux dynamics are not primarily governed by a grass layer. The positive association of GPP with P_intensity_ indicates that the deeper‐rooting tree layer drives CO_2_ dynamics: larger storms infiltrate more deeply (Holdrege et al. 2023), store soil moisture in layers accessed by trees, and thereby enhance tree photosynthesis (Holdrege et al. 2021; Zhang et al. 2016). More years would be needed to conclude if this is a significant relationship.

On the annual scale, P metrics significantly affected ecosystem CO_2_ fluxes at only three savanna sites, likely because aggregation obscures seasonal dynamics. When relationships are evaluated by phenological seasons, more sites show significant associations (Table S11), underscoring the importance of precipitation timing and seasonal variation in soil moisture, temperature, radiation, and litter inputs. Opposing seasonal responses can offset each other and yield negligible annual signals, highlighting the importance of accounting for their concurrent variations on seasonal scales.

### 5.2 Precipitation Intensity and Frequency Are Important for Seasonal CO_2_ Dynamics, Direct and Indirect Effects Differ Across Seasons

The results showed that frequency and intensity of rain events determine precipitation effects on seasonal CO_2_ uptake and release in semi‐arid savannas, beyond overall precipitation amount (Figures 3 and 4). Maximum dry spell length added no unique information to these metrics on the seasonal scale, since it contains similar information as P_frequency_ on inter‐event spacing. The targeted nested model comparisons with LMMs shows that including P_intensity_, P_frequency_, and P_amount_ together maximized model performance. Using P_amount_ alone is therefore not sufficient for explaining seasonal CO_2_ fluxes. Furthermore, the LMMs indicated that the effects of these three P metrics on GPP and R_eco_ strongly differ across phenological seasons.

The SEM results provide deeper insights into differing direct and indirect effects of precipitation metrics on CO_2_ fluxes within the different seasons. Differences between seasons are typically driven by phenological changes as well as differing resource limitations over the year (Nadolski et al. 2025). SWC is the central mediator of precipitation, environmental drivers, and CO_2_ fluxes in most seasons. In the drydown season, Ta is an additional core mediator (Figures 5 and 6).

In the wet season, soil moisture is abundant due to sufficient P_amount_ and leads to increases in GPP, often enhanced by a green grass layer (Figure 5a) (Ardö et al. 2008; Nadolski et al. 2025; Tagesson, Fensholt, Guiro, et al. 2015). However, SWC is limited by SW_in_ and P_intensity_ in this season. Two explanations are possible: (1) with precipitation being packaged into more intense, but fewer events, intervals between events become longer, intensifying plant water stress (Feldman, Feng, et al. 2024) and (2) intense events during the wet season can lead to increased runoff and the water becomes unavailable to plants (Feldman, Feng, et al. 2024). On the other hand, the SEM shows that high P_intensity_ in this season has a significantly positive effect on GPP. This might seem contradicting since P_intensity_ has a suppressing effect on SWC, which would inhibit GPP. However, SWC measurements used here are from the top layer, where SW_in_ leads to evaporation during dry intervals (Scott and Biederman 2019). The direct P_intensity_ effect might be attributed to water drawn from deeper roots by the plants, that is not captured with the top‐layer SWC measurements. P_intensity_ also has a direct positive effect on R_eco_. In a field experiment in a semi‐arid steppe, intense rain events during the growing season lead to an extended increase in soil respiration, with even a single event leading to an elevated soil respiration during the entire growing season (Post and Knapp 2020).

During the drydown season, rainfall decreases, soils dry, and atmospheric dryness rises, leading to the senescence of the grass layer (Luo et al. 2018). In this period, both SWC and Ta become strong mediators of P metrics and CO_2_ fluxes. The SEM results (Figure 5b) indicate that SWC is controlled by P_frequency_ in this season, both directly and via changes in SW_in_ and Ta, which are limited by P_frequency_. With the drydown of the grass layer, SWC loses the effect on GPP, but still enhances R_eco_. With increasing litter (i.e., available carbon coming from the grass layer), increases in SWC can lead to enhanced activity of soil microbes, known as the Birch effect (Birch 1964; Borken and Matzner 2009). Similarly, intense precipitation events enhance R_eco_ in this season. Ta has a suppressing effect on GPP since with increasing temperatures in this season, the heat stress on plants increases.

In the dry season, atmospheric heat and dryness become stronger and soil moisture depletes. The savannas with annual grass layer become mostly dormant in this season, and GPP oftentimes reaches its lowest point (Ma et al. 2020; Tagesson, Fensholt, Cropley, et al. 2015), even if trees are still photosynthesising. If it rains, high P_frequency_ can dim VPD (Figure 5c), and therefore relieve heat and drought stress. If soil moisture is available, it leads to increases in R_eco_, due to the Birch effect and lots of available litter from the senesced grasses and roots (Birch 1964; Huxman, Snyder, et al. 2004; Jarvis et al. 2007). Photodegradation of surface litter (Adair et al. 2017; Berenstecher et al. 2020; Méndez et al. 2019) and microbial mortality during dry periods further have built up decomposable substrates that become available at the first rains and are respirated by microbes (Borken and Matzner 2009; Moyano et al. 2013; Schimel 2018). However, in the dry season, the SEM indicates no direct effects of P metrics on SWC, while SWC affects GPP and R_eco_ significantly (Figure 5c). We suggest that this can be related to soil moisture in deeper soil layers not captured by the measurements used here, that are carried over from antecedent wet seasons (Scott and Biederman 2019) and sustain tree layer GPP throughout parts of the dry season. Furthermore, during night, water vapor adsorption from humidity in the atmosphere into the dry soil can occur in dry ecosystems (Paulus et al. 2025), potentially providing moisture for microbial respiration. While atmospheric humidity has been shown to have effects on the microbial respiration (Wang et al. 2017), the direct connection with soil vapor adsorption is not proven yet to our knowledge and has to be studied further.

The regreening season marks the transition from the late dry season to active growth, beginning with the first rains. On the one hand, when soil moisture crosses the threshold that triggers grass greening and leaf flush for deciduous species (Luo et al. 2020), it leads to increases in GPP (Figure 5d). On the other hand, initial wetting pulses reactivate microbes and rapidly mobilize the labile carbon accumulated during the dry season, generating strong respiration pulses (Jarvis et al. 2007; Reichmann et al. 2013). The SEM results support this pulse‐response framework, with higher SWC increasing R_eco_ (Figure 5d). P_frequency_, rather than P_intensity_ or P_amount_, is the dominant control on SWC in this season. P_frequency_ affects the length of dry intervals, the carryover of residual moisture, and the duration of microbial dormancy. SW_in_ on the contrary, curbs SWC. Overall, the results underscore the central role of SWC in modulating CO_2_ fluxes during the regreening transition.

### 5.3 Limitations

#### 5.3.1 Available Timeseries Length

Semi‐arid savannas have been historically underrepresented in flux research because many regions are remote and lack infrastructure and accessibility due to funding constraints (Bliefernicht et al. 2018; Zhang et al. 2023). Therefore, long‐term measurements from these regions are scarce despite recently growing interest. The flux dataset we compiled here, provides a first global effort to assemble long‐term time series from semi‐arid savannas. Nevertheless, these records of 10 or more years are partly still too short and sparse to detect statistically robust relationships between precipitation variability and CO_2_ fluxes on annual and seasonal scales, underscoring the need for continued long‐term monitoring and broader network coverage.

#### 5.3.2 Seasonal Data Aggregation

The choice of aggregating data by season introduces methodological limitations. For example, when diagnosing the assumption of normality in the LMMs with the Shapiro test, we found the residual distribution to be significantly different from normal in all models (*p* < 0.002). We attribute this to heteroscedasticity in the residuals, that describes an increase in residual variance with increasing flux size in all six models (Figure S12a–f). It can be caused by data aggregation into different group sizes (Schad et al. 2025), which is the case here since the seasons have differing lengths at the different sites in this study.

Another perceived limitation of the seasonal data aggregation used here is that season definitions, although tailored to each site, are held constant across years. In semi‐arid savannas, especially those with an annual grass layer, the start and length of growing and dry seasons varies substantially among years due to a high interannual precipitation variability (Guan et al. 2018; Haverd et al. 2017; Räsänen et al. 2020). However, the aim here is to assess how precipitation variability within specific times of the year affects carbon fluxes. For example, if the onset of rains after the dry period begins earlier than usual and occurs during months designated as dry season, this design keeps the effects of increased precipitation frequency or intensity on carbon fluxes in the dry season. If the end of the dry season were defined each year by rainfall onset, this effect would be masked by dynamic seasons and would not be captured.

#### 5.3.3 Soil Water and Precipitation Measures

In two seasons we observe a stronger effect of P_frequency_ on SWC compared to P_intensity_ and P_amount_ (Figure 5b,d). This might be due to the definition of P days in P_frequency_ starting from 2 mm/day, excluding rain pulses that evaporate right away. Furthermore, we find a positive relationship between SW_in_ and SWC in both drydown and dry season which we cannot fully explain yet. This might be caused by different SWC measurement depths at different sites, with very heterogeneous soil properties (Paulus et al. 2022), or aggregation effects. We would like to point out that soil water potential, measured in situ or estimated via site‐specific pedo‐transfer functions, serves as a more direct indicator of water availability and movement in the soil–plant‐atmosphere continuum in dry ecosystems than SWC, since it directly governs root and microbial functions (Novick et al. 2022; Zhang, Biederman, Schlaepfer, et al. 2025). However, in situ measurements remain scarce and site‐specific pedo‐transfer functions are not available yet at all sites used here. To avoid introducing additional uncertainty via current‐state pedo‐transfer functions, we use SWC in this study. However, incorporating soil water potential in future work would strengthen inference.

This study represents the first compilation of a global semi‐arid savanna flux dataset, and we recommend its continued refinement and expansion.

## 6 Conclusion

This study shows that there are broadly consistent effects of precipitation variability on annual CO_2_ fluxes across global semi‐arid savannas. Increasing precipitation amount, frequency, and intensity mostly lead to both higher gross primary productivity and ecosystem respiration on the annual scale, but with different net impacts on net ecosystem exchange. On the seasonal scale, precipitation frequency and intensity explain more variations in net ecosystem exchange than precipitation amount only. Including precipitation amount, intensity, and frequency together leads to the most improvements using the linear mixed models. Including a metric for dry spell length does not significantly improve the model performance.

Additionally, the seasonal timing of precipitation is crucial, since the direct and indirect effects of precipitation variability on ecosystem CO_2_ fluxes differ according to the phenological seasons. Parsing the year into wet, drydown, dry, and regreening seasons leverages deeper insights into ecosystem processes. Soil water content is the dominant mediator of precipitation‐CO_2_ flux interactions across seasons, with positive effects on both gross primary productivity and ecosystem respiration. In the drydown season, air temperatures are an additional core mediator with a suppressing effect on gross primary productivity.

These findings imply that for accurately representing dryland carbon dynamics in terrestrial biosphere and Earth system models, precipitation amount is not sufficient and we recommend incorporating precipitation frequency and intensity metrics as well as their seasonal timing. To assess how the carbon sink strength of semi‐arid savannas will evolve under more intense but less frequent precipitation events in the future, longer eddy‐covariance time series and robust assessments of regional precipitation trends are essential.

## Author Contributions

**Laura Nadolski:** conceptualization, data curation, formal analysis, investigation, methodology, software, validation, visualization, writing – original draft, writing – review and editing. **Marieke Wesselkamp:** formal analysis, methodology, writing – review and editing. **Tarek El‐Madany:** data curation, supervision. **Markus Lange:** methodology, writing – review and editing. **Jacob Nelson:** conceptualization, supervision, writing – review and editing. **Arnaud Carrara:** data curation, writing – review and editing. **Aleksander Wieckowski:** data curation, writing – review and editing. **Anke Hildebrandt:** writing – review and editing. **Markus Reichstein:** funding acquisition, writing – review and editing. **Sung‐Ching Lee:** conceptualization, funding acquisition, investigation, project administration, resources, supervision, validation, writing – review and editing.

## Conflicts of Interest

The authors declare no conflicts of interest.

## Supporting information


**Table S1:** Definition of seasons at each site.
**Figure S2:** Net ecosystem exchange at ZA‐Kru after marginal distribution gap‐filling with REddyProc.
**Table S3:** *T*‐values for the relationship between precipitation frequency (P_fre) and net ecosystem exchange according to linear mixed effect model output with different thresholds for the definition of rain day. Threshold is shown in mm precipitation. *T*‐values are shown for different seasons.
**Figure S4:** Correlation matrix of Pearson correlation coefficient of seasonal values across all six sites (ES‐LMa, ES‐Abr, US‐Ton, AU‐Dry, AU‐DaS, SN‐Dhr). Red colors show negative correlation, blue colors a positive correlation. The higher the correlation, the darker the color and the bigger the circle depicted in the table.
**Figure S5:** Results of sequential maximum likelihood‐ratio tests with ANOVA. All 24 possible sequences were tested. AIC is the Akaike Information Criterion (lower values indicating better model results). The models m0 describe the null model, m1 contains one precipitation metric, m2 comprises 2 precipitation metrics and so on. The annotation in each bar indicates which metric is added in the respective model. The *p*‐values indicate if the model improved significantly compared to the antecedent model (on its left).
**Figure S6:** Baseline structure of the structural equation models.
**Figure S7:** Relationships between gross primary productivity (GPP, left), ecosystem respiration (R_eco_, right), and (a, b) precipitation amount, (c, d) precipitation frequency (rain days > 2 mm/number of days), (e, f) precipitation intensity (precipitation amount/number of rain days) and (g, h) maximum dry spell length, on the scale of hydrological years across the sites Daly River Savanna (AU‐DaS), Dry River (AU‐Dry), Majadas de Tiétar (ES‐Lma), Tonzi Ranch (US‐Ton), Albuera (ES‐Abr), Great Western Woodlands (AU‐GWW) and Dahra (SN‐Dhr). Solid lines show significant relationships (*p* < 0.05), dotted lines show non‐significant relationships.
**Table S8a:** Output linear mixed effect model with precipitation amount and GPP (Equation 4).
**Table S8b:** Output linear mixed effect model with precipitation amount and R_eco_ (Equation 5).
**Table S8c:** Output linear mixed effect model with precipitation frequency and GPP (Equation 4).
**Table S8d:** Output linear mixed effect model with precipitation frequency and R_eco_ (Equation 5).
**Table S8e:** Output linear mixed effect model with precipitation intensity and GPP (Equation 4).
**Table S8f:** Output linear mixed effect model with precipitation intensity and R_eco_ (Equation 5).
**Figure S8:** Effects of precipitation amount (P_amount_), precipitation frequency (P_frequency_) and precipitation intensity (P_intensity_) on the CO_2_ component fluxes in defined phenological seasons in relation to dry season (direct output of linear mixed effect models, as represented in the Tables S8a–f). They are coloured by significance (*** for *p*‐values < 0.001, ** for *p*‐values < 0.01, * for *p*‐values < 0.05. for *p*‐values < 0.1) which show how the relationship between different metrics of precipitation variability and component fluxes is differs in the defined season compared to the dry season.
**Table S9.1:** Results of post hoc analysis with response variable GPP.
**Table S9.2:** Results of post hoc analysis with response variable R_eco_.
**Table S10.1:** Wet season SEM outputs.
**Table S10.2:** Drydown season SEM outputs.
**Table S10.3:** Dry season SEM outputs.
**Table S10.4:** Regreening season SEM outputs.
**Table S11:** *p*‐values of linear relationships between gross primary productivity (GPP), ecosystem respiration (R_eco_) and net ecosystem exchange (NEE) with precipitation amount (P_am), frequency (P_fre) and intensity (P_int) in different phenological seasons. Dark red shows significantly positive relationships with *p* < 0.05, light red with *p* < 0.1. Dark blue shows significantly negative relationships with *p* < 0.05, light blue with *p* < 0.1.
**Figure S12:** Residuals of the linear mixed effect models. (a) Precipitation amount (P_amount_) model for gross primary productivity (GPP), (b) P_amount_ model for ecosystem respiration (R_eco_), (c) precipitation frequency (P_frequency_) model for GPP, (d) P_frequency_ model for R_eco_, (e) precipitation intensity (P_intensity_) model for GPP, (f) P_intensity_ model for R_eco_.

## Data Availability

The data supporting this study were derived from the following sources, available in the public domain: AU‐GWW & AU‐DaS & AU‐Dry: https://hdl.handle.net/102.100.100/14247, SN‐Dhr: https://doi.org/10.5061/dryad.3ffbg79t0, US‐Ton: https://www.osti.gov/servlets/purl/2204880/, ES‐LMa: https://www.europe‐fluxdata.eu/home/site‐details?id=ES‐LMa, ES‐Abr: https://doi.org/10.5281/zenodo.20589254 (future updated versions of this data can be found later on europe‐fluxdata.eu).
