# Supplementary material for: Impacts of Precipitation Variability on Carbon Flux Dynamics of Global Semi‐Arid Savannas
Source: Glob Chang Biol. 2026 Jun 16;32(6):e70954. doi: 10.1111/gcb.70954 (PMC13270356; doi:10.1111/gcb.70954)
Supplement: Supplementary file 1 — Table S1: Definition of seasons at each site. Figure S2: Net ecosystem exchange at ZA‐Kru after marginal distribution gap‐filling with REddyProc. Table S3: T‐values for the relationship between precipitation frequency (P_fre) and net ecosystem exchange according to linear mixed effect model output with different thresholds for the definition of rain day. Threshold is shown in mm precipitation. T‐values are shown for different seasons. Figure S4: Correlation matrix of Pearson correlation coefficient of seasonal values across all six sites (ES‐LMa, ES‐Abr, US‐Ton, AU‐Dry, AU‐DaS, SN‐Dhr). Red colors show negative correlation, blue colors a positive correlation. The higher the correlation, the darker the color and the bigger the circle depicted in the table. Figure S5: Results of sequential maximum likelihood‐ratio tests with ANOVA. All 24 possible sequences were tested. AIC is the Akaike Information Criterion (lower values indicating better model results). The models m0 describe the null model, m1 contains one precipitation metric, m2 comprises 2 precipitation metrics and so on. The annotation in each bar indicates which metric is added in the respective model. The p‐values indicate if the model improved significantly compared to the antecedent model (on its left). Figure S6: Baseline structure of the structural equation models. Figure S7: Relationships between gross primary productivity (GPP, left), ecosystem respiration (Reco, right), and (a, b) precipitation amount, (c, d) precipitation frequency (rain days > 2 mm/number of days), (e, f) precipitation intensity (precipitation amount/number of rain days) and (g, h) maximum dry spell length, on the scale of hydrological years across the sites Daly River Savanna (AU‐DaS), Dry River (AU‐Dry), Majadas de Tiétar (ES‐Lma), Tonzi Ranch (US‐Ton), Albuera (ES‐Abr), Great Western Woodlands (AU‐GWW) and Dahra (SN‐Dhr). Solid lines show significant relationships (p < 0.05), dotted lines show non‐significant relationships. Table [file GCB-32-e70954-s001.docx]

Supplementary material

**S.1: Seasons**

**Table S1**: Definition of seasons at each site.

| **Fluxnet ID** | **Wet Season** | **Drydown** | **Dry season** | **Regreening** |
| --- | --- | --- | --- | --- |
| ES-Lma | Dec-April | May-June | July-Sep | Oct-Nov |
| ES-Abr | Dec-April | May-June | July-Sep | Oct-Nov |
| US-Ton | Dec-April | May-June | July-Oct | Nov |
| ZA-Kru | Dec-Feb | March-May | June-Sep | Oct-Nov |
| SN-Dhr | Aug-Sep | Oct-Dec | Jan-June | July |
| AU-Dry | Jan-March | April-May | June-Sep | Oct-Dec |
| AU-DaS | Jan-March | April-May | June-Sep | Oct-Dec |

**S.2: Skukuza (ZA-Kru) flux data**

**Processing details**

The raw flux data from ZA-Kru was processed with Eddysoft since the beginning of the flux tower installation in 2001 until end of 2013. Since 11/12/2013 data was processed with EddyPro. The data has been cleaned visually, outranged values (due to instrument failure, as well as sensor default values) have been eliminated. Quality control has been applied and quality-flagging was adapted from Mauder and Foken et al (2004). The post-processing was conducted with REddyProc v. 1.3.3 (Wutzler et al., 2018). The u*-thresholds were estimated for different seasons, starting in the months 3, 6, 10 and 12 (compare Table S1) (Papale et al., 2006; Wutzler et al., 2018). Gaps were filled with marginal distribution sampling (Reichstein et al., 2005). NEE was supposed to be partitioned into GPP and R_eco_ using night-time partitioning method as implemented in REddyProc (Wutzler et al., 2018), as it was also conducted at the other sites. However, the gaps in the T_air_ timeseries were too big to partition in a reasonable way.


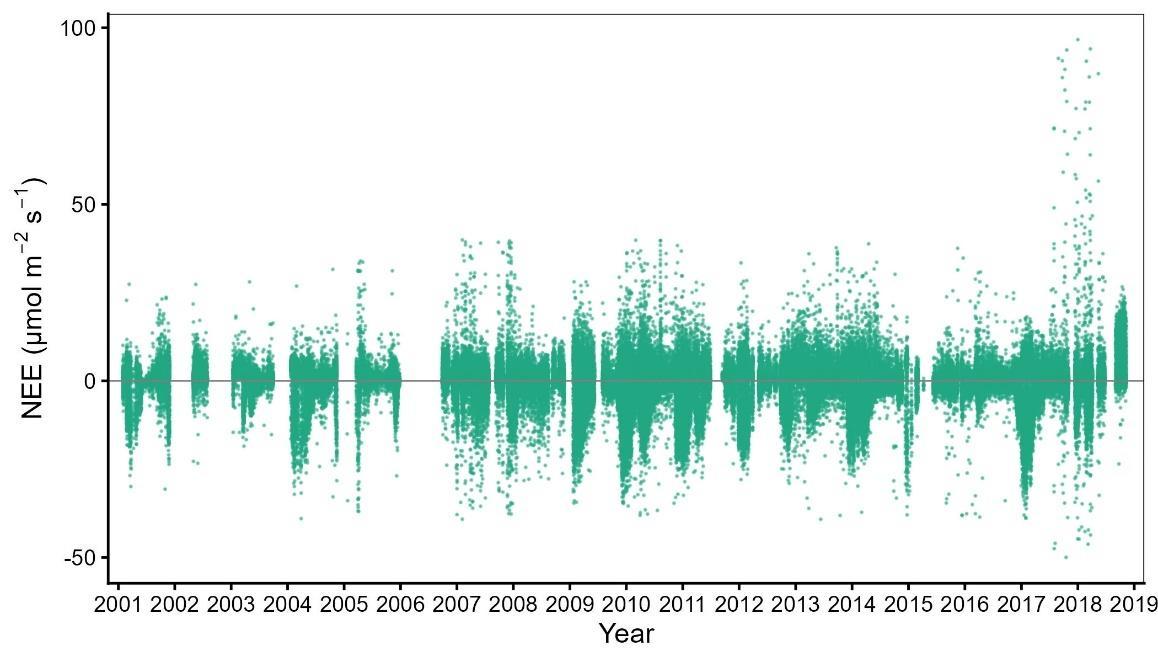


**Figure S2**: Net ecosystem exchange at ZA-Kru after marginal distribution gap-filling with REddyProc.

**S.3: Results of sensitivity analysis of linear mixed model output to precipitation frequency threshold**


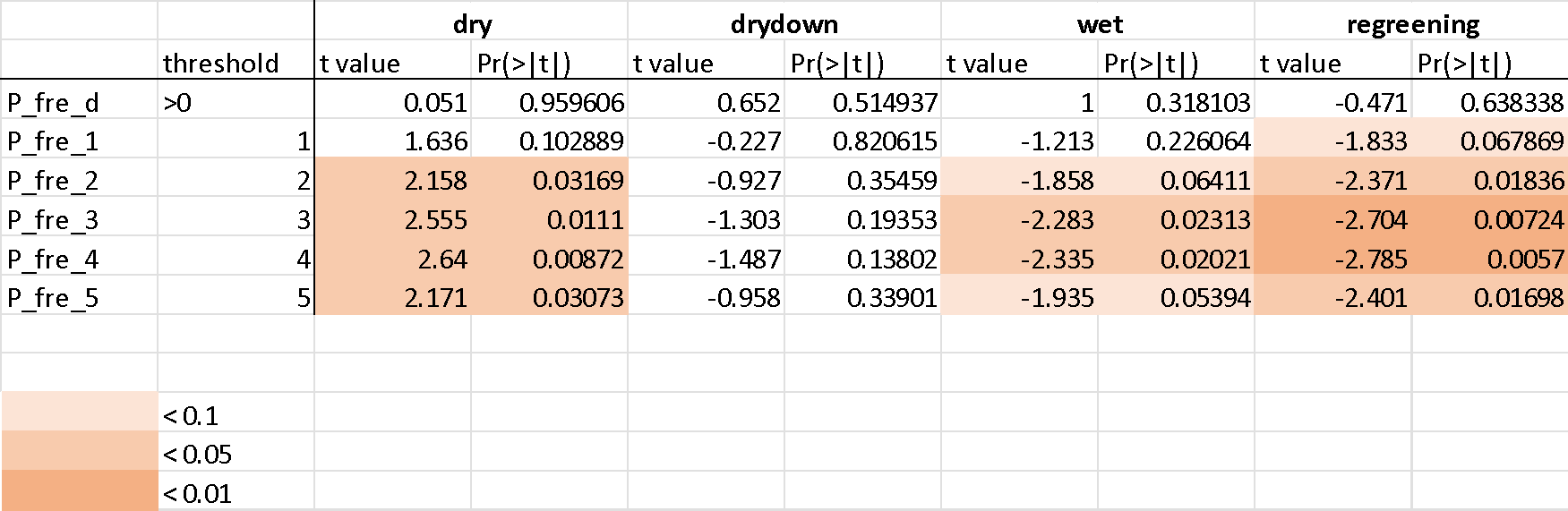


**Table S3**: T-values for the relationship between precipitation frequency (P_fre) and net ecosystem exchange according to linear mixed effect model output with different thresholds for the definition of rain day. Threshold is shown in mm precipitation. T-values are shown for different seasons.

**S.4: Correlation matrix**


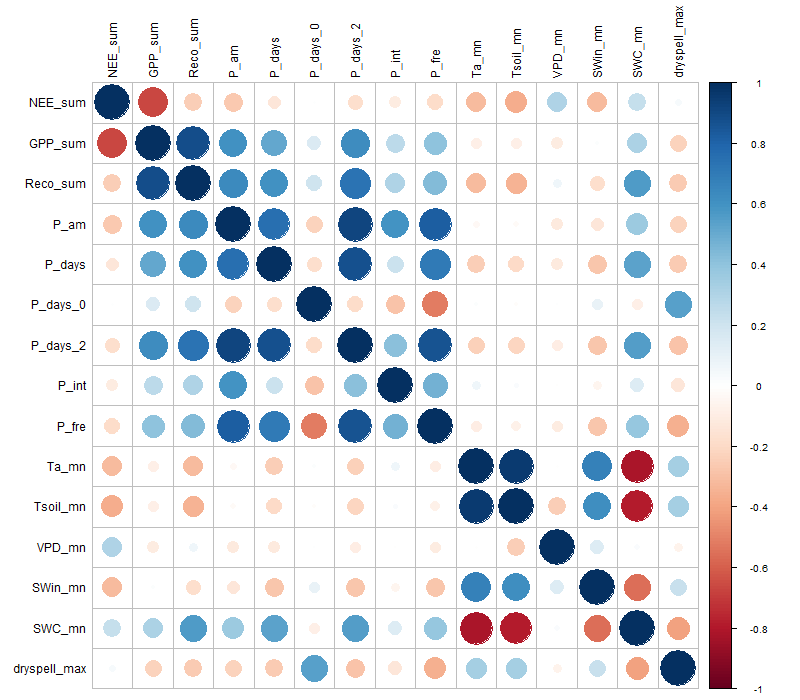


**Figure S4**: Correlation matrix of Pearson correlation coefficient of seasonal values across all six sites (ES-LMa, ES-Abr, US-Ton, AU-Dry, AU-DaS, SN-Dhr). Red colors show negative correlation, blue colors a positive correlation. The higher the correlation, the darker the color and the bigger the circle depicted in the table.

**S5: Model outputs of targeted nested model comparison**

**
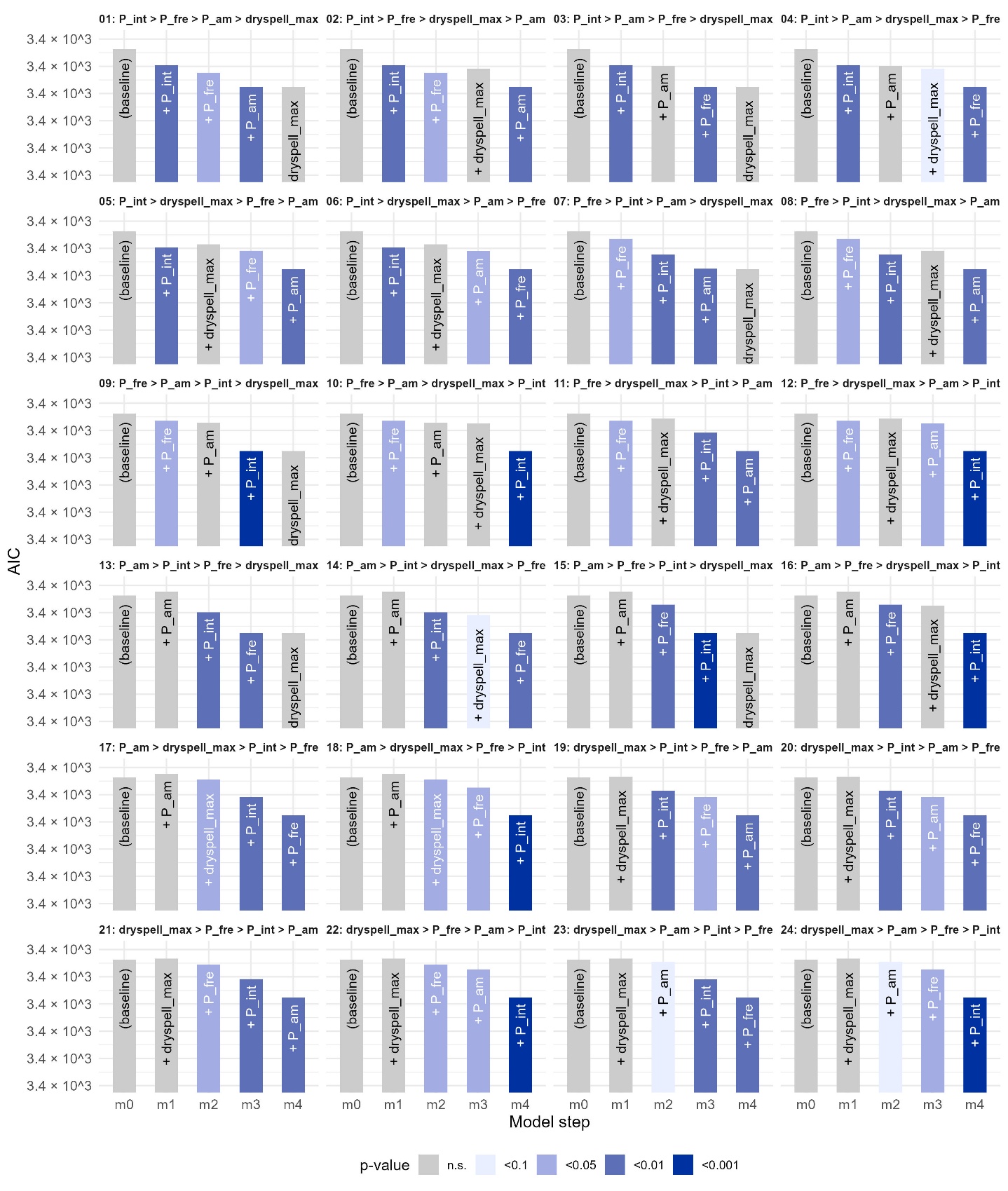
**

**Fig. S5:** Results of sequential maximum likelihood-ratio tests with ANOVA. All 24 possible sequences were tested. AIC is the Akaike Information Criterion (lower values indicating better model results). The models m0 describe the null model, m1 contains one precipitation metric, m2 comprises 2 precipitation metrics and so on. The annotation in each bar indicates which metric is added in the respective model. The p-values indicate if the model improved significantly compared to the antecedent model (on its left).

**S.6: Baseline structural equation model**

**
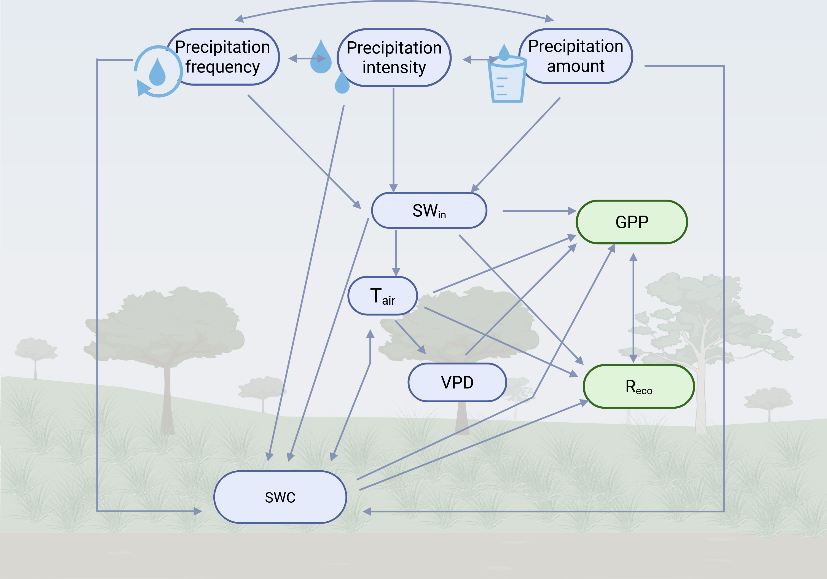
**

**Figure S6**: Baseline structure of the structural equation models

**S.7: Relationships of gross primary productivity and ecosystem respiration with precipitation metrics**


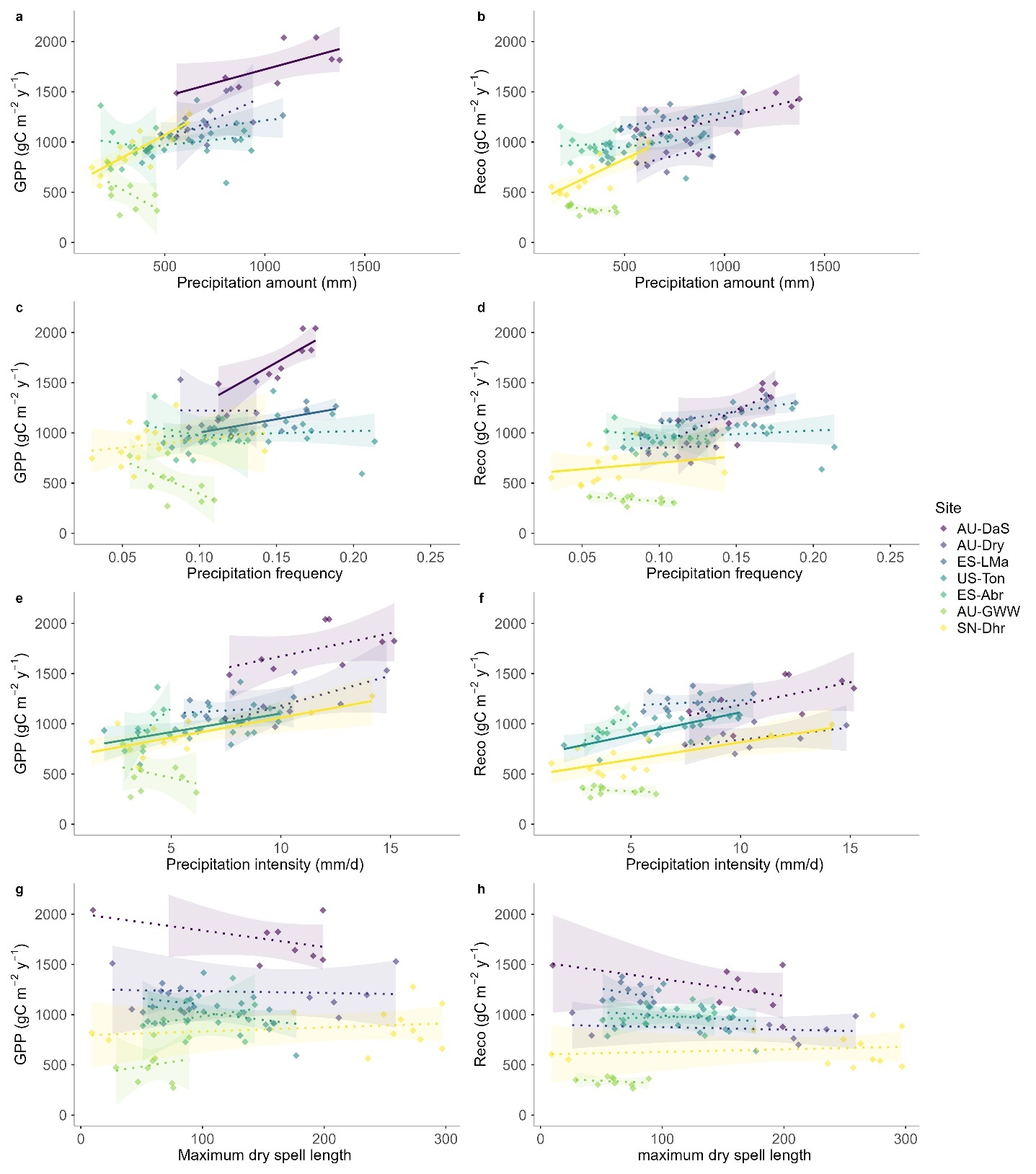


**Fig. S7**: Relationships between gross primary productivity (GPP, left), ecosystem respiration (R_eco_, right), and (a-b) precipitation amount, (c-d) precipitation frequency (rain days > 2mm/number of days), (e-f) precipitation intensity (precipitation amount/number of rain days) and (g-h) maximum dry spell length, on the scale of hydrological years across the sites Daly River Savanna (AU-DaS), Dry River (AU-Dry), Majadas de Tiétar (ES-Lma), Tonzi Ranch (US-Ton), Albuera (ES-Abr), Great Western Woodlands (AU-GWW) and Dahra (SN-Dhr). Solid lines show significant relationships (p < 0.05), dotted lines show non-significant relationships.

**S.8: Results of linear mixed effect models**

The P_amount_-specific LMM for GPP shows that in the dry season, the P_amount_ slope is small and not significant. Relative to the dry season, none of the season-specific P_amount_ slopes differ (all interaction terms non-significant) (Table S8a). These estimates imply the following season-specific slopes while holding other covariates constant: dry: +17.9, drydown: −43.2, regreening: +34.1, wet: +14.5 (none statistically different from the dry season). Among other predictors, GPP increases with SWin and decreases with Ta. Intercepts are lower in regreening and higher in the wet season relative to the dry season. In contrast, P_amount_ strongly affects R_eco_ in the dry season, with higher P_amount_ associated with higher R_eco_ (Table S8b). Relative to the dry season, the P_amount_ slope does not differ in drydown, but is significantly lower in regreening and wet seasons. These contrasts imply the following season-specific P_amount_ slopes while holding other covariates constant: dry: +150.6, drydown: +72.4, regreening: +35.6 (different from dry), wet: +8.5 (different from dry). R_eco_ also increases with SWC and SWin, shows a marginally negative Tsoil × SWC interaction, and has a negative main effect of Tsoil. Intercepts are lower in drydown and regreening relative to the dry season.

In the P_frequency_-specific LMM, the P_frequency_ slope with GPP is small and not significant in the dry season (reference) (Table S8c). Relative to the dry season, the P_frequency_ slope does not differ in drydown, regreening, or wet season. These estimates imply the following season-specific P_frequency_ slopes while holding other covariates constant: dry: +17.5, drydown: +6.6, regreening: +24.6, wet: +9.6 (none statistically different from the dry season). Among other predictors, GPP increases with SWin and decreases with Ta; VPD and SWC show no clear main effects. The intercept is marginally lower in regreening (p = 0.056) and higher in the wet season (p < 0.001) relative to dry season. Contrastingly, P_frequency_ strongly predicts R_eco_ in the dry season, with higher P_frequency_ associated with higher R_eco_ (Table S8d). Relative to the dry season, the P_frequency_ slope is significantly lower in drydown, regreening, and wet season. The season-specific P_frequency_ slopes while holding other covariates constant are: dry: +139.8, drydown: +38.8, regreening: +10.1, wet: −5.4. R_eco_ also increases with SWC and SWin, decreases with Tsoil, and shows a negative Tsoil × SWC interaction. Intercepts are lower in drydown and regreening relative to the dry season, with no difference in wet.

The P_intensity_-specific model shows that the P_intensity_ slope with GPP is small and not significant in the dry season (Table S8e). Relative to the dry season, the P_intensity_ slope does not differ in drydown or regreening, but is significantly higher in the wet season. These estimates imply the following season-specific P_intensity_ slopes while holding other covariates constant: dry: +6.6, drydown: −7.1, regreening: +17.8, wet: +43.4. Among other predictors, GPP increases with SWin (p < 0.001) and decreases with Ta, while VPD and SWC show no clear main effects. Intercepts are lower in regreening and higher in the wet season relative to dry; drydown does not differ significantly from dry. P_intensity_ positively affects R_eco_ in the dry season (Table S8f). Relative to the dry season, the P_intensity_ slope does not differ in drydown, regreening, or wet. The season-specific P_intensity_ slopes while holding other covariates constant are: dry: +22.1, drydown: +11.7, regreening: +13.9, wet: +13.9 (none statistically different from dry). R_eco_ also increases with SWC and SWin, decreases with Tsoil, and shows a negative Tsoil × SWC interaction. Intercepts are lower in drydown and regreening and higher in the wet season relative to dry season. The relationships between precipitation and CO_2_ component fluxes are season‑dependent and differ by precipitation metric.

**Table S8a**. Output linear mixed effect model with precipitation amount and GPP (Eq 4).

| **Random effects** | |  |  |  |  |
| --- | --- | --- | --- | --- | --- |
| **grp** | **var1** | **var2** | **vcov** | **sdcor** |  |
| year:Site | (Intercept) | | 729.4077 | 27.00755 |  |
| year:Site | SWC_mn |  | 408.2494 | 20.20518 |  |
| year:Site | (Intercept) | SWC_mn | -97.8063 | -0.17923 |  |
| Site | (Intercept) | | 7459.037 | 86.36572 |  |
| Site | SWC_mn |  | 3887.773 | 62.35201 |  |
| Site | (Intercept) | SWC_mn | -2992.03 | -0.55561 |  |
| Residual |  |  | 3260.319 | 57.0992 |  |
|  |  |  |  |  |  |
| **Fixed effects** | |  |  |  |  |
| **term** | **Estimate** | **Std..Error** | **df** | **t.value** | **Pr...t..** |
| (Intercept) | 254.5996 | 54.23532 | 17.36457 | 4.69435 | 0.000198 |
| Ta_mn | -49.2341 | 15.52432 | 78.65032 | -3.17142 | 0.002163 |
| SWC_mn | 53.5004 | 30.49959 | 8.329884 | 1.754135 | 0.116001 |
| SWin_mn | 66.14982 | 8.352465 | 197.5739 | 7.919796 | 1.69E-13 |
| VPD_mn | -17.8394 | 15.61783 | 39.75069 | -1.14225 | 0.260185 |
| seasondrydown | -42.2066 | 47.68986 | 233.2558 | -0.88502 | 0.377055 |
| seasonregreening | -99.2414 | 39.92157 | 233.6866 | -2.48591 | 0.013622 |
| seasonwet | 217.1525 | 43.09185 | 220.6736 | 5.039294 | 9.72E-07 |
| P_am | 17.93912 | 56.45444 | 225.9501 | 0.317763 | 0.750958 |
| seasondrydown:P_am | -61.1068 | 69.88693 | 228.0262 | -0.87437 | 0.382839 |
| seasonregreening:P_am | 16.20104 | 57.38184 | 224.252 | 0.282337 | 0.777945 |
| seasonwet:P_am | -3.39055 | 57.04309 | 228.4797 | -0.05944 | 0.952655 |
|  |  |  |  |  |  |
| **AIC** | **BIC** | **logLik** | **deviance** | **df_residual** |  |
| 3202.636 | 3271.697 | -1582.32 | 3164.636 | 261 |  |

**Table S8b**. Output linear mixed effect model with precipitation amount and R_eco_ (Eq 5).

| **Random effects** | |  |  |  |  |
| --- | --- | --- | --- | --- | --- |
| **grp** | **var1** | **var2** | **vcov** | **sdcor** |  |
| year:Site | (Intercept) | | 296.5145 | 17.2196 |  |
| Site | (Intercept) | | 2549.616 | 50.49373 |  |
| Residual |  |  | 2330.326 | 48.27345 |  |
|  |  |  |  |  |  |
| **Fixed effects** | |  |  |  |  |
| **term** | **Estimate** | **Std..Error** | **df** | **t.value** | **Pr...t..** |
| (Intercept) | 329.5493 | 37.53582 | 48.23882 | 8.779594 | 1.44E-11 |
| Tsoil_mn | -34.5537 | 12.39343 | 172.5977 | -2.78806 | 0.005897 |
| SWC_mn | 42.49887 | 12.2368 | 258.7497 | 3.473039 | 0.000603 |
| seasondrydown | -79.0596 | 38.66282 | 260.2044 | -2.04485 | 0.041876 |
| seasonregreening | -182.214 | 31.53527 | 256.7789 | -5.77811 | 2.18E-08 |
| seasonwet | 30.39722 | 33.47984 | 258.631 | 0.907926 | 0.364763 |
| P_am | 150.5526 | 44.97088 | 257.5814 | 3.347779 | 0.000936 |
| SWin_mn | 27.27803 | 6.644906 | 219.6736 | 4.105104 | 5.7E-05 |
| Tsoil_mn:SWC_mn | -13.2999 | 6.933207 | 238.3949 | -1.91828 | 0.056269 |
| seasondrydown:P_am | -78.1527 | 56.93868 | 259.9194 | -1.37258 | 0.171067 |
| seasonregreening:P_am | -114.965 | 46.02825 | 253.4498 | -2.49771 | 0.013134 |
| seasonwet:P_am | -142.097 | 45.40255 | 257.7508 | -3.12971 | 0.001951 |
|  |  |  |  |  |  |
| **AIC** | **BIC** | **logLik** | **deviance** | **df_residual** |  |
| 2938.572 | 2992.548 | -1454.29 | 2908.572 | 255 |  |

**Table S8c**. Output linear mixed effect model with precipitation frequency and GPP (Eq 4).

| **Random effects** | |  |  |  |  |
| --- | --- | --- | --- | --- | --- |
| **grp** | **var1** | **var2** | **vcov** | **sdcor** |  |
| year:Site | (Intercept) | | 819.7376 | 28.63106 |  |
| year:Site | SWC_mn |  | 338.6104 | 18.40137 |  |
| year:Site | (Intercept) | SWC_mn | -67.052 | -0.12727 |  |
| Site | (Intercept) | | 6930.21 | 83.24789 |  |
| Site | SWC_mn |  | 4212.842 | 64.90641 |  |
| Site | (Intercept) | SWC_mn | -3614.92 | -0.66902 |  |
| Residual |  |  | 3364.386 | 58.00332 |  |
|  |  |  |  |  |  |
| **Fixed effects** | |  |  |  |  |
| **term** | **Estimate** | **Std..Error** | **df** | **t.value** | **Pr...t..** |
| (Intercept) | 247.2066 | 64.70352 | 37.28833 | 3.820605 | 0.000489 |
| Ta_mn | -45.4277 | 15.96931 | 88.91881 | -2.84468 | 0.005517 |
| SWC_mn | 46.95026 | 31.15113 | 7.667405 | 1.507177 | 0.171807 |
| SWin_mn | 70.82397 | 8.631685 | 206.3388 | 8.205116 | 2.46E-14 |
| VPD_mn | -16.8733 | 16.09874 | 29.82191 | -1.04811 | 0.303008 |
| seasondrydown | -14.664 | 55.24342 | 232.9129 | -0.26544 | 0.790902 |
| seasonregreening | -104.567 | 54.45871 | 232.2844 | -1.92012 | 0.056067 |
| seasonwet | 229.2574 | 58.15763 | 234.6743 | 3.942001 | 0.000107 |
| P_fre | 17.50034 | 57.46774 | 229.4409 | 0.304525 | 0.761004 |
| seasondrydown:P_fre | -10.9224 | 59.45297 | 230.6526 | -0.18372 | 0.854398 |
| seasonregreening:P_fre | 7.121825 | 58.15784 | 229.0654 | 0.122457 | 0.902645 |
| seasonwet:P_fre | -7.9213 | 57.77663 | 229.5333 | -0.1371 | 0.89107 |
|  |  |  |  |  |  |
| **AIC** | **BIC** | **logLik** | **deviance** | **df_residual** |  |
| 3209.302 | 3278.363 | -1585.65 | 3171.302 | 261 |  |

**Table S8d**. Output linear mixed effect model with precipitation frequency and R_eco_ (Eq 5).

| **Random effects** | |  |  |  |  |
| --- | --- | --- | --- | --- | --- |
| **grp** | **var1** | **var2** | **vcov** | **sdcor** |  |
| year:Site | (Intercept) | | 440.9888 | 20.99973 |  |
| Site | (Intercept) | | 3152.285 | 56.14522 |  |
| Residual |  |  | 2348.133 | 48.45754 |  |
|  |  |  |  |  |  |
| **Fixed effects** | |  |  |  |  |
| **term** | **Estimate** | **Std..Error** | **df** | **t.value** | **Pr...t..** |
| (Intercept) | 350.4434 | 46.31117 | 64.03674 | 7.567146 | 1.86E-10 |
| Tsoil_mn | -28.4724 | 13.17609 | 182.1682 | -2.16091 | 0.032008 |
| SWC_mn | 41.15017 | 13.35008 | 258.7704 | 3.082392 | 0.002275 |
| seasondrydown | -124.989 | 41.62951 | 244.4745 | -3.00242 | 0.002956 |
| seasonregreening | -208.544 | 40.95859 | 248.4371 | -5.09158 | 7.02E-07 |
| seasonwet | 17.6439 | 43.51146 | 246.7524 | 0.4055 | 0.685461 |
| P_fre | 139.8422 | 43.5979 | 250.3123 | 3.207544 | 0.001513 |
| SWin_mn | 31.12897 | 6.724393 | 218.8688 | 4.629261 | 6.29E-06 |
| Tsoil_mn:SWC_mn | -20.1449 | 7.373363 | 247.1737 | -2.73212 | 0.006747 |
| seasondrydown:P_fre | -100.997 | 45.54663 | 246.2724 | -2.21743 | 0.027507 |
| seasonregreening:P_fre | -129.775 | 43.92007 | 247.4325 | -2.95481 | 0.00343 |
| seasonwet:P_fre | -145.221 | 43.84653 | 249.4224 | -3.31204 | 0.001063 |
|  |  |  |  |  |  |
| **AIC** | **BIC** | **logLik** | **deviance** | **df_residual** |  |
| 2951.223 | 3005.199 | -1460.61 | 2921.223 | 255 |  |

**Table S8e**. Output linear mixed effect model with precipitation intensity and GPP (Eq 4).

| **Random effects** | |  |  |  |  |
| --- | --- | --- | --- | --- | --- |
| **grp** | **var1** | **var2** | **vcov** | **sdcor** |  |
| year:Site | (Intercept) | | 666.6678 | 25.81991 |  |
| year:Site | SWC_mn |  | 398.0286 | 19.95065 |  |
| year:Site | (Intercept) | SWC_mn | -165.35 | -0.32099 |  |
| Site | (Intercept) | | 5797.128 | 76.13887 |  |
| Site | SWC_mn |  | 6183.928 | 78.63795 |  |
| Site | (Intercept) | SWC_mn | -4178.55 | -0.69789 |  |
| Residual |  |  | 3249.574 | 57.00504 |  |
|  |  |  |  |  |  |
| **Fixed effects** | |  |  |  |  |
| **term** | **Estimate** | **Std..Error** | **df** | **t.value** | **Pr...t..** |
| (Intercept) | 230.8555 | 35.39995 | 5.203627 | 6.521352 | 0.001088 |
| Ta_mn | -43.5396 | 14.77974 | 84.2748 | -2.9459 | 0.004163 |
| SWC_mn | 47.16806 | 35.69594 | 6.395963 | 1.321384 | 0.231675 |
| SWin_mn | 70.52528 | 8.213284 | 196.7271 | 8.586733 | 2.71E-15 |
| VPD_mn | -15.8028 | 14.81305 | 16.74756 | -1.06682 | 0.301191 |
| seasondrydown | -9.6594 | 12.90359 | 179.7902 | -0.74858 | 0.455087 |
| seasonregreening | -91.3759 | 15.23429 | 162.3312 | -5.99804 | 1.26E-08 |
| seasonwet | 227.7244 | 20.18881 | 68.31861 | 11.27974 | 2.96E-17 |
| P_int | 6.556154 | 11.24371 | 229.9952 | 0.583095 | 0.5604 |
| seasondrydown:P_int | -13.6921 | 13.50569 | 222.9054 | -1.0138 | 0.311777 |
| seasonregreening:P_int | 11.27609 | 14.02005 | 218.3442 | 0.804283 | 0.422108 |
| seasonwet:P_int | 36.80785 | 16.07837 | 226.8574 | 2.289277 | 0.022983 |
|  |  |  |  |  |  |
| **AIC** | **BIC** | **logLik** | **deviance** | **df_residual** |  |
| 3198.461 | 3267.522 | -1580.23 | 3160.461 | 261 |  |

**Table S8f**. Output linear mixed effect model with precipitation intensity and R_eco_ (Eq 5).

| **Random effects** | |  |  |  |  |
| --- | --- | --- | --- | --- | --- |
| **grp** | **var1** | **var2** | **vcov** | **sdcor** |  |
| year:Site | (Intercept) | | 304.1566 | 17.44009 |  |
| Site | (Intercept) | | 2882.377 | 53.68778 |  |
| Residual |  |  | 2458.665 | 49.58493 |  |
|  |  |  |  |  |  |
| **Fixed effects** | |  |  |  |  |
| **term** | **Estimate** | **Std..Error** | **df** | **t.value** | **Pr...t..** |
| (Intercept) | 237.3734 | 25.20921 | 9.016351 | 9.416138 | 5.82E-06 |
| Tsoil_mn | -30.8365 | 12.78065 | 181.6806 | -2.41275 | 0.016827 |
| SWC_mn | 44.13269 | 12.34284 | 262.8516 | 3.575572 | 0.000416 |
| seasondrydown | -32.3683 | 11.0172 | 228.2532 | -2.93798 | 0.003642 |
| seasonregreening | -94.4013 | 12.55558 | 236.6213 | -7.51867 | 1.14E-12 |
| seasonwet | 119.9949 | 15.43051 | 246.0641 | 7.776467 | 2.04E-13 |
| P_int | 22.06207 | 9.262705 | 260.4289 | 2.381817 | 0.017946 |
| SWin_mn | 32.11805 | 6.747747 | 219.2154 | 4.759818 | 3.52E-06 |
| Tsoil_mn:SWC_mn | -18.2692 | 7.092758 | 242.7052 | -2.57576 | 0.010595 |
| seasondrydown:P_int | -10.3618 | 11.36197 | 256.9473 | -0.91197 | 0.362639 |
| seasonregreening:P_int | -8.17898 | 11.78782 | 242.5703 | -0.69385 | 0.48844 |
| seasonwet:P_int | -8.15754 | 13.29848 | 251.5723 | -0.61342 | 0.540154 |
|  |  |  |  |  |  |
| **AIC** | **BIC** | **logLik** | **deviance** | **df_residual** |  |
| 2952.855 | 3006.832 | -1461.43 | 2922.855 | 255 |  |


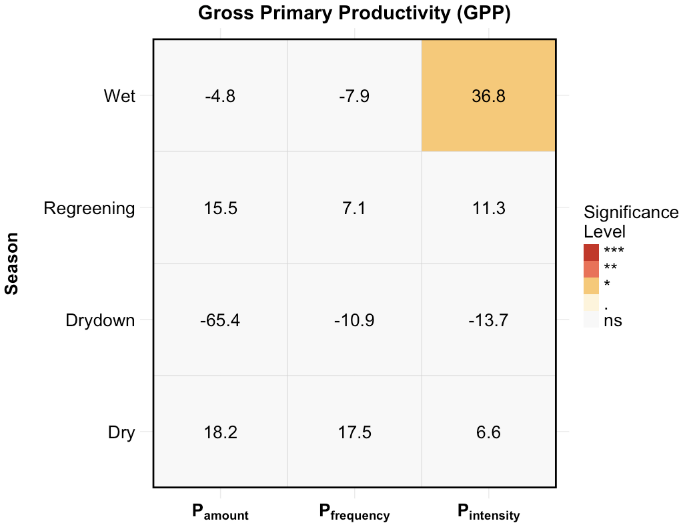

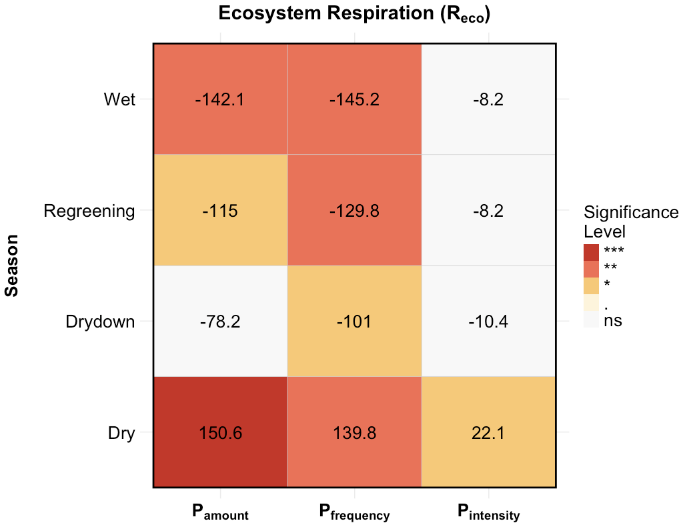


**Fig. S8:** Effects of precipitation amount (P_amount_), precipitation frequency (P_frequency_) and precipitation intensity (P_intensity_) on the CO_2_ component fluxes in defined phenological seasons in relation to dry season (direct output of linear mixed effect models, as represented in the Tables S8a-f). They are coloured by significance (*** stands for p-values < 0.001, ** for p-values < 0.01, * for p-values < 0.05, . for p-values < 0.1) which show how the relationship between different metrics of precipitation variability and component fluxes is differs in the defined season compared to the dry season.

**S.9: Simple Slopes Analysis with emtrends (emmeans R package)**

To decompose the significant interactions between precipitation variability and season, we conducted a simple slope post-hoc analysis using the emtrends function from the emmeans package (Lenth and Piaskowski, 2026) applied to each LMM. For each P metric, we estimated the marginal slope of the predictor on GPP or Reco within each season, holding all other continuous predictors constant at their mean. These season-specific slopes represent the total conditional effect of each P metric, computed as the linear combination of the main effect coefficient and the corresponding interaction coefficient (β_main + β_interaction). Standard errors were derived from the variance-covariance matrix of the fixed effects, accounting for the covariance between the main effect and interaction coefficients. Degrees of freedom were approximated using the Kenward-Roger method. We tested whether each seasonal slope differed significantly from zero using a t-test, and the resulting p-values are visualised in Figure 3 in the main document.

**Table S9.1:** Results of post-hoc analysis with response variable GPP.

Model: P_amount

| season | P_am.trend | SE | df | lower.CL | upper.CL | t.ratio | p.value |
| --- | --- | --- | --- | --- | --- | --- | --- |
| dry | 18.2 | 58.5 | 228 | -96.97 | 133.4 | 0.312 | 0.7556 |
| drydown | -47.2 | 44.7 | 214 | -135.22 | 40.9 | -1.055 | 0.2924 |
| regreening | 33.7 | 12.2 | 231 | 9.69 | 57.7 | 2.766 | 0.0061 |
| wet | 13.4 | 11.1 | 122 | -8.58 | 35.4 | 1.207 | 0.2297 |

Model: P_frequency

| season | P_fre.trend | SE | df | lower.CL | upper.CL | t.ratio | p.value |
| --- | --- | --- | --- | --- | --- | --- | --- |
| dry | 17.50 | 59.8 | 248 | -100.225 | 135.2 | 0.293 | 0.7699 |
| drydown | 6.58 | 24.4 | 235 | -41.461 | 54.6 | 0.270 | 0.7876 |
| regreening | 24.62 | 12.4 | 256 | 0.212 | 49.0 | 1.986 | 0.0481 |
| wet | 9.58 | 12.3 | 255 | -14.718 | 33.9 | 0.776 | 0.4382 |

Model: P_intensity

| season | P_int.trend | SE | df | lower.CL | upper.CL | t.ratio | p.value |
| --- | --- | --- | --- | --- | --- | --- | --- |
| dry | 6.56 | 11.70 | 248 | -16.397 | 29.51 | 0.563 | 0.5742 |
| drydown | -7.14 | 8.05 | 235 | -23.002 | 8.73 | -0.886 | 0.3765 |
| regreening | 17.83 | 9.19 | 243 | -0.274 | 35.94 | 1.940 | 0.0535 |
| wet | 43.36 | 13.40 | 225 | 16.955 | 69.77 | 3.236 | 0.0014 |

**Table S9.2:** Results of post-hoc analysis with response variable R_eco_.

Model: P_amount

| season | P_am.trend | SE | df | lower.CL | upper.CL | t.ratio | p.value |
| --- | --- | --- | --- | --- | --- | --- | --- |
| dry | 150.55 | 46.40 | 271 | 59.24 | 241.9 | 3.246 | 0.0013 |
| drydown | 72.40 | 37.30 | 266 | -1.10 | 145.9 | 1.940 | 0.0535 |
| regreening | 35.59 | 9.88 | 273 | 16.13 | 55.0 | 3.601 | 0.0004 |
| wet | 8.46 | 7.53 | 268 | -6.37 | 23.3 | 1.123 | 0.2624 |

Model: P_frequency

| season | P_fre.trend | SE | df | lower.CL | upper.CL | t.ratio | p.value |
| --- | --- | --- | --- | --- | --- | --- | --- |
| dry | 139.84 | 45.20 | 265 | 50.80 | 228.9 | 3.092 | 0.0022 |
| drydown | 38.85 | 21.30 | 265 | -3.01 | 80.7 | 1.827 | 0.0688 |
| regreening | 10.07 | 10.20 | 275 | -9.94 | 30.1 | 0.991 | 0.3227 |
| wet | -5.38 | 9.71 | 269 | -24.50 | 13.7 | -0.554 | 0.5801 |

Model: P_intensity

| season | P_int.trend | SE | df | lower.CL | upper.CL | t.ratio | p.value |
| --- | --- | --- | --- | --- | --- | --- | --- |
| dry | 22.1 | 9.57 | 274 | 3.23 | 40.9 | 2.306 | 0.0219 |
| drydown | 11.7 | 6.79 | 268 | -1.67 | 25.1 | 1.723 | 0.0861 |
| regreening | 13.9 | 7.81 | 273 | -1.49 | 29.3 | 1.778 | 0.0764 |
| wet | 13.9 | 10.30 | 271 | -6.46 | 34.3 | 1.344 | 0.1799 |

**S.10: Path coefficients and model diagnostics of structural equation models**

**Table S10.1**: Wet season SEM outputs

Coefficients:

Response Predictor Estimate Std.Error DF Crit.Value P.Value Std.Estimate

SWin_mn P_am -0.0324 0.0182 59 -1.7839 0.0796 -0.1321

SWin_mn P_fre 3.5344 24.4874 59 0.1443 0.8857 0.0066

SWin_mn P_int 0.3756 0.7781 59 0.4827 0.6311 0.0284

Ta_mn SWin_mn 0.0316 0.0066 61 4.7921 0.0000 0.1921

VPD_mn Ta_mn 2.6915 4.5681 61 0.5892 0.5579 0.1443

SWC_mn SWin_mn -0.1102 0.0241 58 -4.5672 0.0000 -0.5505

SWC_mn P_am 0.0156 0.0044 58 3.5579 0.0008 0.3177

SWC_mn P_fre -0.4579 5.7768 58 -0.0793 0.9371 -0.0043

SWC_mn P_int -0.5448 0.1842 58 -2.9575 0.0045 -0.2061

GPP_sum VPD_mn -0.0522 0.24 56 -0.2174 0.8287 -0.0774

GPP_sum SWin_mn -0.2729 0.9916 56 -0.2752 0.7842 -0.1319

GPP_sum SWC_mn 10.5085 4.6037 56 2.2826 0.0263 1.0162

GPP_sum Ta_mn 7.8333 8.3741 56 0.9354 0.3536 0.6228

GPP_sum P_am -0.2024 0.1141 56 -1.7740 0.0815 -0.3988

GPP_sum P_int 21.0708 5.4842 56 3.8421 0.0003 0.7710

Reco_sum Ta_mn 1.3153 5.2975 57 0.2483 0.8048 0.1042

Reco_sum SWC_mn 5.2383 2.9136 57 1.7979 0.0775 0.5045

Reco_sum SWin_mn -1.1276 0.7359 57 -1.5323 0.1310 -0.5427

Reco_sum P_am -0.0500 0.092 57 -0.5436 0.5888 -0.0981

Reco_sum P_int 13.7154 4.5756 57 2.9975 0.0040 0.4998

~~P_am ~~P_fre 0.6875 - 66 7.6918 0.0000 0.6875

~~P_am ~~P_int 0.8112 - 66 11.2705 0.0000 0.8112

~~P_int ~~P_fre 0.6048 - 66 6.1693 0.0000 0.6048

~~GPP_sum ~~Reco_sum 0.7918 - 68 10.4530 0.0000 0.7918

~~Ta_mn ~~SWC_mn -0.1595 - 68 -1.3025 0.0987 -0.1595

---

Individual R-squared:

Response method Marginal Conditional

SWin_mn none 0.01 0.97

Ta_mn none 0.05 0.99

VPD_mn none 0.01 0.98

SWC_mn none 0.58 0.91

GPP_sum none 0.17 0.79

Reco_sum none 0.56 0.69

Fisher's C = 20.513 with P-value = 0.551 and on 22 degrees of freedom

**Table S10.2**: Drydown season SEM outputs

Coefficients:

Response Predictor Estimate Std.Error DF Crit.Value P.Value Std.Estimate

SWin_mn P_am -0.1334 0.0918 59 -1.4534 0.1514 -0.1092

SWin_mn P_fre -145.2013 52.4194 59 -2.7700 0.0075 -0.1319

SWin_mn P_int 0.8871 0.5157 59 1.7202 0.0906 0.0993

Ta_mn SWin_mn -0.0398 0.0086 60 -4.6016 0.0000 -0.5788

Ta_mn P_fre -18.3480 3.6384 60 -5.0429 0.0000 -0.2425

VPD_mn Ta_mn 28.3519 9.4441 61 3.0021 0.0039 0.1719

SWC_mn SWin_mn 0.0496 0.0156 58 3.1854 0.0023 0.4892

SWC_mn P_am 0.0057 0.0232 58 0.2433 0.8086 0.0456

SWC_mn P_fre 48.5577 12.9166 58 3.7593 0.0004 0.4349

SWC_mn P_int -0.0043 0.1297 58 -0.0334 0.9735 -0.0048

GPP_sum VPD_mn -0.0507 0.0538 58 -0.9436 0.3493 -0.3214

GPP_sum SWin_mn 0.6527 0.4973 58 1.3124 0.1945 0.3647

GPP_sum SWC_mn 5.1612 3.5875 58 1.4387 0.1556 0.2925

GPP_sum Ta_mn -20.6712 6.0462 58 -3.4189 0.0012 -0.7938

Reco_sum Ta_mn -10.1697 5.5 58 -1.8490 0.0696 -0.4490

Reco_sum SWC_mn 7.0177 2.3617 58 2.9715 0.0043 0.4572

Reco_sum SWin_mn -0.0802 0.4001 58 -0.2006 0.8417 -0.0515

Reco_sum P_int 2.4542 1.1855 58 2.0702 0.0429 0.1764

~~P_am ~~P_fre 0.7260 - 66 8.5774 0.0000 0.7260

~~P_am ~~P_int 0.8313 - 66 12.1514 0.0000 0.8313

~~P_int ~~P_fre 0.3926 - 66 3.4674 0.0009 0.3926

~~GPP_sum ~~Reco_sum 0.8074 - 68 11.0335 0.0000 0.8074

~~Ta_mn ~~SWC_mn -0.3225 - 68 -2.7467 0.0039 -0.3225

---

Individual R-squared:

Response method Marginal Conditional

SWin_mn none 0.03 0.97

Ta_mn none 0.66 0.86

VPD_mn none 0.02 0.99

SWC_mn none 0.59 0.75

GPP_sum none 0.40 0.94

Reco_sum none 0.38 0.83

Fisher's C = 22.79 with P-value = 0.645 and on 26 degrees of freedom

**Table S10.3**: Dry season SEM outputs

Coefficients:

Response Predictor Estimate Std.Error DF Crit.Value P.Value Std.Estimate

SWin_mn P_am -0.1426 0.077 55 -1.8520 0.0694 -0.2327

SWin_mn P_fre 37.0328 95.5277 55 0.3877 0.6998 0.0398

SWin_mn P_int 1.0741 0.4781 55 2.2467 0.0287 0.2190

Ta_mn SWin_mn -0.0228 0.0101 57 -2.2672 0.0272 -0.1969

VPD_mn Ta_mn 12.4370 8.7927 56 1.4145 0.1628 0.0359

VPD_mn P_fre -718.7778 349.1911 56 -2.0584 0.0442 -0.0192

SWC_mn SWin_mn 0.0223 0.0129 54 1.7313 0.0891 0.1875

SWC_mn P_am 0.0124 0.008 54 1.5483 0.1274 0.1705

SWC_mn P_fre -2.1100 9.6679 54 -0.2182 0.8281 -0.0191

SWC_mn P_int 0.0500 0.0504 54 0.9909 0.3262 0.0857

GPP_sum VPD_mn -0.0396 0.0663 54 -0.5966 0.5533 -0.3013

GPP_sum SWin_mn 0.4774 0.9238 54 0.5167 0.6075 0.0905

GPP_sum SWC_mn 27.0137 13.3767 54 2.0195 0.0484 0.6086

GPP_sum Ta_mn -1.6628 10.6677 54 -0.1559 0.8767 -0.0365

Reco_sum Ta_mn -8.4013 5.7087 52 -1.4717 0.1471 -0.2938

Reco_sum SWC_mn 13.6495 4.6416 52 2.9407 0.0049 0.4898

Reco_sum SWin_mn -0.4330 0.4943 52 -0.8759 0.3851 -0.1308

Reco_sum P_am 0.0989 0.2959 52 0.3342 0.7396 0.0487

Reco_sum P_fre 273.4885 343.1676 52 0.7970 0.4291 0.0888

Reco_sum P_int 1.8411 1.8146 52 1.0146 0.3150 0.1133

~~P_am ~~P_fre 0.7400 - 62 8.6639 0.0000 0.7400

~~P_am ~~P_int 0.7893 - 62 10.1229 0.0000 0.7893

~~P_int ~~P_fre 0.5084 - 62 4.6481 0.0000 0.5084

~~GPP_sum ~~Reco_sum 0.7233 - 64 8.1808 0.0000 0.7233

~~Ta_mn ~~SWC_mn 0.0053 - 64 0.0414 0.4836 0.0053

---

Individual R-squared:

Response method Marginal Conditional

SWin_mn none 0.01 0.87

Ta_mn none 0.04 0.92

VPD_mn none 0.00 1.00

SWC_mn none 0.14 0.85

GPP_sum none 0.11 0.92

Reco_sum none 0.28 0.90

Fisher's C = 22.058 with P-value = 0.456 and on 22 degrees of freedom

**Table S10.4**: Regreening season SEM outputs

Coefficients:

Response Predictor Estimate Std.Error DF Crit.Value P.Value Std.Estimate

SWin_mn P_am -0.0149 0.0224 60 -0.6674 0.5070 -0.0344

SWin_mn P_fre -40.0391 21.6577 60 -1.8487 0.0694 -0.0466

SWin_mn P_int -0.2731 0.4895 60 -0.5580 0.5789 -0.0169

Ta_mn SWin_mn 0.0231 0.0093 62 2.4906 0.0154 0.2143

VPD_mn Ta_mn 3.3405 7.0598 62 0.4732 0.6377 0.1183

SWC_mn SWin_mn -0.0719 0.0137 59 -5.2394 0.0000 -0.7882

SWC_mn P_am -0.0033 0.0069 59 -0.4829 0.6310 -0.0837

SWC_mn P_fre 18.0606 7.5252 59 2.4000 0.0196 0.2308

SWC_mn P_int 0.1124 0.1634 59 0.6878 0.4943 0.0761

GPP_sum VPD_mn -0.0281 0.049 59 -0.5728 0.5690 -0.0524

GPP_sum SWin_mn 0.2794 0.3547 59 0.7878 0.4340 0.1714

GPP_sum SWC_mn 20.5929 9.2534 59 2.2254 0.0299 1.1512

GPP_sum Ta_mn -5.0710 3.4186 59 -1.4834 0.1433 -0.3353

Reco_sum Ta_mn 2.1719 4.0585 60 0.5351 0.5945 0.2164

Reco_sum SWC_mn 5.2708 1.1014 60 4.7857 0.0000 0.4440

Reco_sum SWin_mn -0.1289 0.3491 60 -0.3691 0.7133 -0.1191

~~P_am ~~P_fre 0.5382 - 67 5.2271 0.0000 0.5382

~~P_am ~~P_int 0.6683 - 67 7.3545 0.0000 0.6683

~~P_int ~~P_fre 0.1951 - 67 1.6283 0.1082 0.1951

~~GPP_sum ~~Reco_sum 0.3423 - 69 2.9598 0.0021 0.3423

~~Ta_mn ~~SWC_mn -0.3833 - 69 -3.3715 0.0006 -0.3833

---

Individual R-squared:

Response method Marginal Conditional

SWin_mn none 0.01 0.98

Ta_mn none 0.06 0.98

VPD_mn none 0.01 0.95

SWC_mn none 0.62 0.70

GPP_sum none 0.19 0.99

Reco_sum none 0.10 0.88

Fisher's C = 42.816 with P-value = 0.061 and on 30 degrees of freedom

**S.11: Seasonal precipitation – CO_2_ flux relationships.**

**Table S11:** P-values of linear relationships between gross primary productivity (GPP), ecosystem respiration (R_eco_) and net ecosystem exchange (NEE) with precipitation amount (P_am), frequency (P_fre) and intensity (P_int) in different phenological seasons. Dark red shows significantly positive relationships with p< 0.05, light red with p< 0.1. Dark blue shows significantly negative relationships with p< 0.05, light blue with p<0.1.

| **wet** |  |  |  |  |  |  |  |  |  |
| --- | --- | --- | --- | --- | --- | --- | --- | --- | --- |
|  | GPP |  |  | Reco |  |  | NEE |  |  |
| site | P_am | P_fre | P_int | P_am | P_fre | P_int | P_am | P_fre | P_int |
| ES-Abr | 0.660 | 0.870 | 0.290 | 0.770 | 0.930 | 0.250 | 0.520 | 0.790 | 0.370 |
| SN-Dhr | 0.000 | 0.570 | 0.001 | 0.001 | 0.620 | 0.002 | 0.002 | 0.430 | 0.010 |
| US-Ton | 0.140 | 0.460 | 0.079 | 0.010 | 0.028 | 0.002 | 0.280 | 0.100 | 0.190 |
| ES-LMa | 0.690 | 0.430 | 0.960 | 0.530 | 0.340 | 0.890 | 0.520 | 0.640 | 0.560 |
| AU-Dry | 0.630 | 0.200 | 0.270 | 0.790 | 0.320 | 0.350 | 0.190 | 0.071 | 0.240 |
| AU-DaS | 0.076 | 0.060 | 0.120 | 0.160 | 0.140 | 0.180 | 0.250 | 0.290 | 0.690 |
|  |  |  |  |  |  |  |  |  |  |
|  |  |  |  |  |  |  |  |  |  |
| **drydown** |  |  |  |  |  |  |  |  |  |
|  | GPP |  |  | Reco |  |  | NEE |  |  |
| site | P_am | P_fre | P_int | P_am | P_fre | P_int | P_am | P_fre | P_int |
| ES-Abr | 0.750 | 0.210 | 0.900 | 0.240 | 0.034 | 0.400 | 0.360 | 0.950 | 0.370 |
| SN-Dhr | 0.330 | 0.340 | 0.360 | 0.810 | 0.600 | 0.860 | 0.082 | 0.230 | 0.078 |
| US-Ton | 0.290 | 0.170 | 0.032 | 0.400 | 0.380 | 0.008 | 0.650 | 0.290 | 0.570 |
| ES-LMa | 0.800 | 0.220 | 0.890 | 0.008 | 0.015 | 0.071 | 0.120 | 0.670 | 0.059 |
| AU-Dry | 0.960 | 0.710 | 0.940 | 0.970 | 0.740 | 0.680 | 0.930 | 0.840 | 0.840 |
| AU-DaS | 0.360 | 0.280 | 0.940 | 0.710 | 0.600 | 0.230 | 0.015 | 0.240 | 0.050 |
|  |  |  |  |  |  |  |  |  |  |
|  |  |  |  |  |  |  |  |  |  |
| **dry** |  |  |  |  |  |  |  |  |  |
|  | GPP |  |  | Reco |  |  | NEE |  |  |
| site | P_am | P_fre | P_int | P_am | P_fre | P_int | P_am | P_fre | P_int |
| ES-Abr | 0.780 | 0.580 | 0.780 | 0.490 | 0.930 | 0.810 | 0.280 | 0.230 | 0.300 |
| SN-Dhr | 0.920 | 0.330 | 0.670 | 0.065 | 0.630 | 0.240 | 0.240 | 0.043 | 0.680 |
| US-Ton | 0.120 | 0.150 | 0.470 | 0.290 | 0.180 | 0.001 | 0.210 | 0.420 | 0.110 |
| ES-LMa | 0.400 | 0.990 | 0.330 | 0.060 | 0.150 | 0.270 | 0.420 | 0.059 | 0.880 |
| AU-Dry | 0.320 | 0.930 | 0.230 | 0.520 | 0.920 | 0.360 | 0.360 | 0.850 | 0.330 |
| AU-DaS | 0.320 | 0.180 | 0.140 | 0.140 | 0.053 | 0.060 | 0.780 | 0.610 | 0.730 |
|  |  |  |  |  |  |  |  |  |  |
|  |  |  |  |  |  |  |  |  |  |
| **regreening** | |  |  |  |  |  |  |  |  |
|  | GPP |  |  | Reco |  |  | NEE |  |  |
| site | P_am | P_fre | P_int | P_am | P_fre | P_int | P_am | P_fre | P_int |
| ES-Abr | 0.330 | 0.320 | 0.036 | 0.260 | 0.950 | 0.360 | 0.850 | 0.073 | 0.051 |
| SN-Dhr | 0.096 | 0.250 | 0.290 | 0.019 | 0.250 | 0.120 | 0.540 | 0.390 | 0.750 |
| US-Ton | 0.012 | 0.002 | 0.900 | 0.001 | 0.003 | 0.740 | 0.049 | 0.300 | 0.580 |
| ES-LMa | 0.290 | 0.900 | 0.250 | 0.170 | 0.630 | 0.230 | 1.000 | 0.740 | 0.780 |
| AU-Dry | 0.410 | 0.310 | 0.210 | 0.700 | 0.810 | 0.880 | 0.082 | 0.250 | 0.130 |
| AU-DaS | 0.820 | 0.590 | 0.710 | 0.290 | 0.034 | 0.650 | 0.710 | 0.520 | 0.940 |

**S.12: Plots of residuals of linear mixed effect models**

**
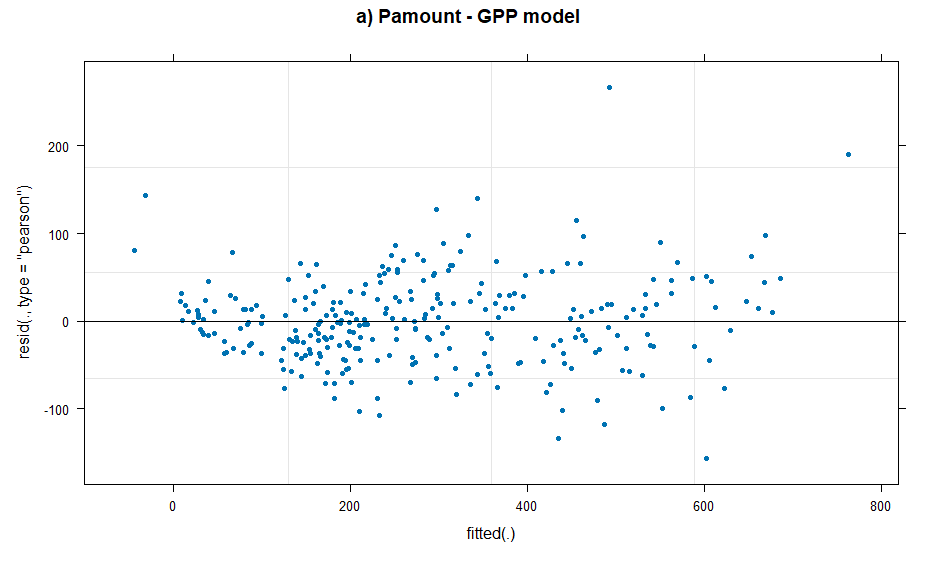
**

**
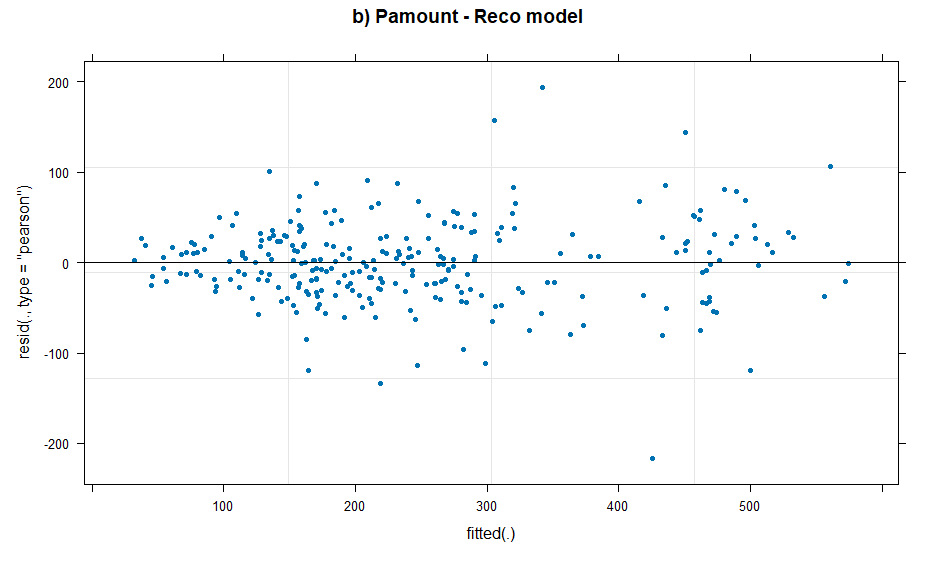
**

**
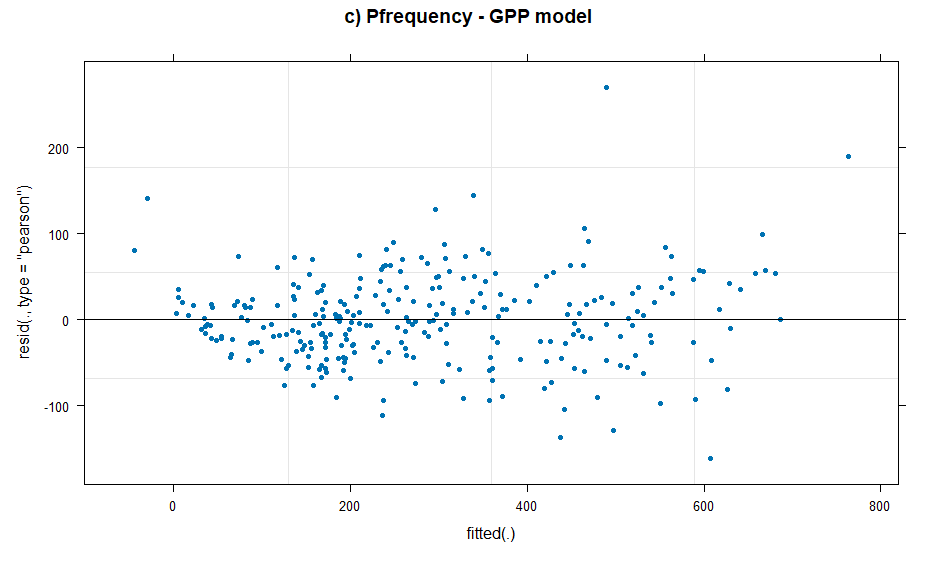
**

**
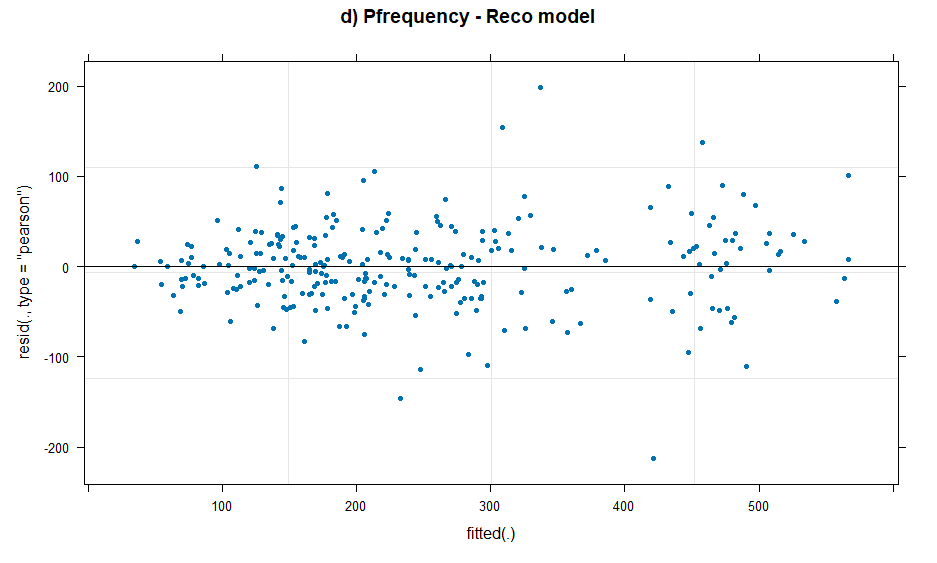
**

**
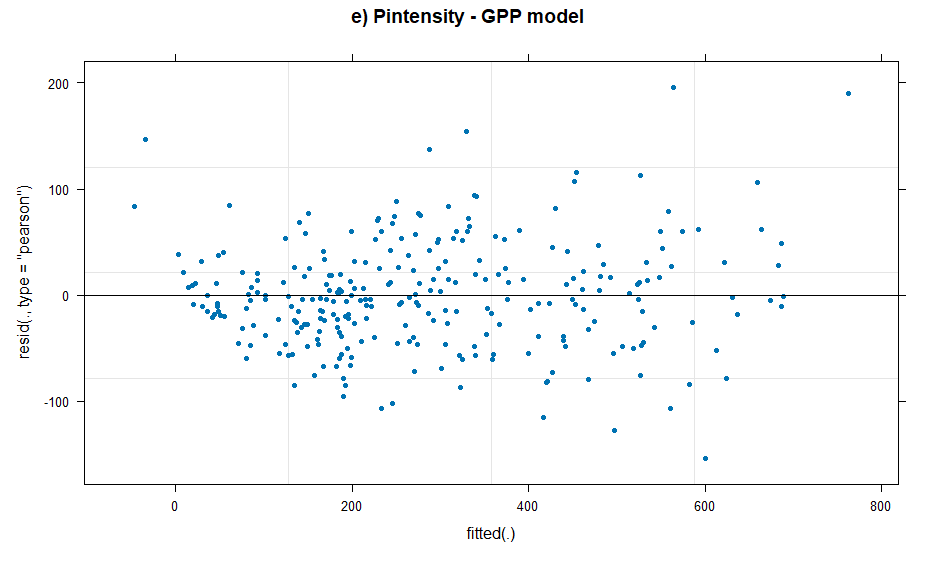
**

**
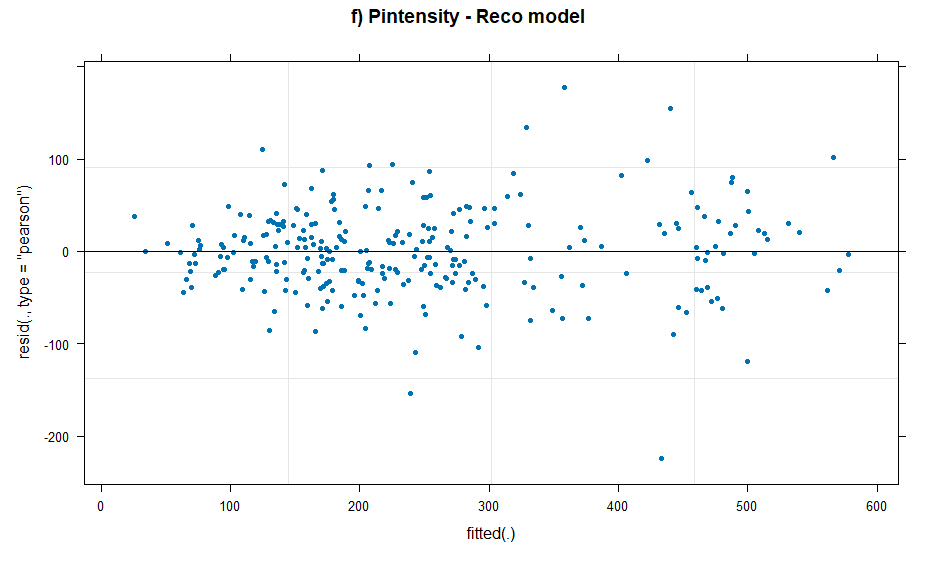
**

**Fig. S12:** Residuals of the linear mixed effect models. a) Precipitation amount (P_amount_) model for gross primary productivity (GPP), b) P_amount_ model for ecosystem respiration (Reco), c) precipitation frequency (P_frequency_) model for GPP, d) P_frequency_ model for R_eco_, e) precipitation intensity (P_intensity_) model for GPP, f) P_intensity_ model for R_eco_.
